# Nose‐to‐Brain Delivery of *Chlorella vulgaris* Extracellular Vesicles for Antidepressant Effects

**DOI:** 10.1002/jev2.70198

**Published:** 2025-11-18

**Authors:** Kangyu Jin, Ruoxi Wang, Bing Chen, Danni Zhong, Shangping Cheng, Aiying Tong, Yangjian Qi, Jing Lu, Min Zhou

**Affiliations:** ^1^ Eye Center, The Second Affiliated Hospital Zhejiang University School of Medicine Hangzhou China; ^2^ Institute of Translational Medicine Zhejiang University Hangzhou China; ^3^ Department of Psychiatry, the First Affiliated Hospital Zhejiang University School of Medicine Hangzhou China; ^4^ Zhejiang Key Laboratory of Precision Psychiatry Hangzhou China; ^5^ Zhejiang University‐University of Edinburgh Institute (ZJU‐UoE Institute), Zhejiang University School of Medicine Zhejiang University Haining China; ^6^ Songjiang Research Institute Songjiang Hospital Affiliated to Shanghai Jiao Tong University School of Medicine Shanghai China; ^7^ Zhejiang University‐Ordos City Etuoke Banner Joint Research Center Haining China

**Keywords:** blood–brain barrier, Chlorella‐derived extracellular vesicles, intranasal administration, nose‐to‐brain pathway, rapid antidepressant

## Abstract

Current antidepressants face limitations due to the blood–brain barrier (BBB), systemic side effects and delayed onset. Here, we engineered an intranasal thermosensitive hydrogel (EVs@IN) encapsulating *Chlorella vulgaris*‐derived extracellular vesicles (EVs) for sustained nose‐to‐brain delivery. EVs@IN significantly enhanced nasal mucosal retention and facilitated targeted transport of EVs to the hippocampus via olfactory pathways, while minimizing pulmonary exposure and clearance. In mouse models of depression (LPS‐induced and CUMS), intranasal EVs@IN elicited rapid and potent alleviation of depressive‐ and anxiety‐like behaviours. Mechanistically, EVs modulated astrocyte phenotypic transformation, reducing the release of neurotoxic complement C3 and suppressing neuroinflammation. Concurrently, they activated the Nrf2‐Pgc‐1α pathway, enhanced antioxidant defences (elevated SOD and GSH), mitigated oxidative stress and restored synaptic plasticity and neurogenesis in the hippocampus. Furthermore, we demonstrated the capacity of EVs to serve as efficient drug carriers for brain delivery. EVs@IN exhibited excellent long‐term biocompatibility in vivo. Our findings establish plant‐derived EVs within a sustained‐release intranasal platform as a promising, scalable and BBB‐bypassing strategy for the rapid treatment of depression and potentially other neuropsychiatric disorders.

## Graphical abstract

A schematic illustration of *Chlorella vulgaris*‐derived extracellular vesicles (EVs) for brain‐targeted delivery and their antidepressant mechanisms. This diagram depicts how an intranasal thermosensitive hydrogel (EVs@IN) encapsulating *Chlorella vulgaris*‐derived EVs achieves rapid antidepressant effects by modulating astrocyte phenotypic transformation, reducing neurotoxic complement C3 release and suppressing neuroinflammation.

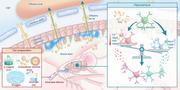

## 1 Introduction

Depression is a common and disabling mental disorder affecting millions of individuals worldwide (C.‐M. D. Collaborators 2021; Weisler et al. 2016). The morbidity and mortality associated with major depression render it the number one cause of disability worldwide and exert an extraordinary economic burden on society in terms of lost productivity (Ding et al. 2016). Current clinical interventions face dual challenges: while antidepressants and psychotherapy demonstrate therapeutic efficacy, many patients experience delayed treatment response (2–4 weeks) and insufficient symptom remission (Perlis 2016). Furthermore, first‐line medications frequently induce adverse effects, including weight gain and sexual dysfunction, with relapse risk within 2 years of discontinuation (Cui et al. 2024). Therefore, it is crucial to develop antidepressants that act quickly and have few side effects.

The treatment of depression is challenged by the blood–brain barrier (BBB) and the blood‐cerebrospinal barrier that prevent the crossing of drugs from the systemic circulation into the central nervous system (Liang et al. 2022). While increasing the dose of the drug to achieve the desired intracranial therapeutic effect may lead to dose‐dependent adverse reactions, especially effects on liver and kidney function, because increased drug doses are distributed non‐selectively throughout the body's organ systems. Naso‐brain drug delivery is a non‐invasive method of drug delivery. Nasal delivery can bypass the BBB and deliver drugs directly to the brain (Wu et al. 2023). However, there are many problems with nasal administration, including the limited volume of the nasal cavity and the retention time, which further reduces the residence time of drugs in the nasal cavity and affects the efficiency of drugs entering the brain (Agrawal et al. 2018; Dighe et al. 2023). There are also many metabolic enzymes in the nasal mucosa (Khatri et al. 2023). Therefore, reducing the degradation of drugs in the nasal cavity and increasing their residence time are essential for effective nasal administration of drugs to the brain.

Extracellular vesicles (EVs) are lipid‐bilayer particles naturally released by a diverse range of cells, from microorganisms and plants to human cells (Akers et al. 2013). They play key roles in intercellular communication by transferring encapsulated bioactive molecules such as DNA, RNA, proteins and lipids (Kahroba et al. 2019; Raposo and Stoorvogel 2013). EVs derived from human cells have shown considerable therapeutic potential in previous studies (Sul et al. 2024; Prieto‐Vila et al. 2024; Wiklander et al. 2024). Yet their clinical translation is hampered by the challenge of scalable production. In contrast, plant‐derived EVs have attracted significant interest due to their ease of production and demonstrated bioactivity (Wang et al. 2024; You et al. 2021). *Chlorella vulgaris*, in particular, is rich in vitamins, phenolic compounds and polysaccharides with known anti‐inflammatory and antioxidant properties. It also contains several bioactive components that support energy metabolism and mitochondrial biogenesis (Heo et al. 2026). Moreover, CV extracts have been reported to alleviate somatic and cognitive symptoms related to depression and anxiety (Panahi et al. 2015). Therefore, EVs derived from CV represent a promising therapeutic agent with great application potential.

In this study, we encapsulated EVs within an inulin gel (EVs@IN), which addresses the aforementioned limitations by enhancing mucosal adhesion and enabling controlled release. This formulation effectively reduces the risk of coughing induced by direct nasal administration while ensuring efficient delivery to the brain. EVs@IN adheres strongly to the nasal mucosa, prolongs residence time, avoids rapid clearance by nasal mucociliary action and facilitates the release of EVs for transport to the brain via the olfactory nerve pathways. Furthermore, we validated its mechanism of action: EVs@IN regulates reactive astrocyte phenotypes, reduces complement C3 release, activates the Nrf2‐Pgc‐1α pathway, enhances SOD and GSH production and alleviates neuroinflammation, thereby improving depression‐like behaviours. Overall, EVs@IN represents a promising strategy for treating depression.

## 2 Materials and Methods

Key resources are provided in Table S1.

### 2.1 Materials

CV and BG11 medium were obtained from Guangyu Biotechnology (Shanghai, China). Inulin was purchased from Psaitong Biotechnology (Beijing, China). All other reagents were of analytical grade and used as received.

### 2.2 Preparation of EVs and EVs@IN

CV was cultured in BG11 medium under standard conditions. Extracellular vesicles (EVs) were isolated via sequential ultracentrifugation and concentrated to 2 mg/mL (protein concentration determined by BCA assay) in PBS. IN was prepared by dissolving 3 g of inulin in 10 mL of water at 45°C, followed by gelation at 4°C overnight. Equal volumes of EVs and preformed IN were blended via dropwise addition under sonication and incubated at 37°C for 2 h to form EVs@IN.

### 2.3 Characterization of EVs and EVs@IN

The morphology of EVs was characterized using transmission electron microscopy (Talos 120 kV, Thermo Fisher). The total protein content of EVs was quantified using the bicinchoninic acid (BCA) assay (Beyotime, China). To assess reproducibility, three independent batches were prepared and characterized. Particle size and zeta potential of EVs and EVs@IN were measured by dynamic light scattering (Zetasizer Nano‐ZS90, Malvern Instruments). The rheological properties of EVs@IN were evaluated with an Anton Paar MCR 302 rheometer using frequency sweep and viscosity measurements. The storage stability and injectability of EVs@IN were assessed at 4°C over 7 days.

### 2.4 In Vitro Release Assay

Release kinetics of EVs from EVs@IN were assessed using a Transwell system (Nest, China) with phosphate‐buffered saline (PBS, Servicebio, China) in the lower chamber. At defined time points (0.5–72 h), aliquots were collected and replenished with fresh medium. Protein concentration was quantified by BCA assay to calculate cumulative release.

### 2.5 In Vivo Fluorescence Imaging and Biodistribution

Male C57BL/6J mice (6 weeks old, *n* = 3 per group) were administered fluorescently labelled EVs (PKH26 kit, Solarbio, China) or EVs@IN intranasally (20 µL; EVs = 2 mg/mL, IN = 150 mg/mL). Control groups administered with an equivalent dose of free PKH26 dye or PKH26@IN hydrogel were included and processed identically. Free dye was removed by ultracentrifugation prior to use. At multiple time points (0.5–72 h), mice were anesthetized and imaged using the IVIS Lumina LT Series III system (PerkinElmer, USA). Total radiant efficiency was analyzed using Living Image 4.5 software. At each time point, mice were sacrificed and major organs (brain, heart, liver, spleen, lungs, kidneys, GI tract) were harvested for ex vivo imaging. Faeces were collected for metabolic tracking.

### 2.6 FD4 Loading Into EVs and Characterization

To prepare FD4‐loaded EVs, 0.3 mg of EVs was incubated with FD4 (0.6–4.8 mg) in PBS under passive or sonication‐assisted conditions. For sonication, samples underwent six 30‐s cycles at 20 kHz and 150 W with 2‐min cooling intervals on ice. All samples were incubated at 4°C for 12 h post‐loading. Free FD4 was removed by ultracentrifugation (100,000 × *g*, 2 h, 4°C). A standard curve was constructed by preparing a series of FD4 solutions at known concentrations (0.03125, 0.0625, 0.125, 0.25, 0.5 and 1 mg/mL) and measuring their absorbance at 495 nm. The resulting calibration curve exhibited a high correlation coefficient (*R* ^2^ > 0.99), ensuring accurate quantification. The concentration of free FD4 in the supernatant was determined by measuring its absorbance at 495 nm and interpolating from the standard curve. The encapsulation efficiency (EE) was calculated as EE (%) = (Total FD4 amount—Free FD4 amount)/Total FD4 amount × 100%.

### 2.7 Animals

All animal procedures were approved by the Animal Experimental Ethical Committee of the First Affiliated Hospital, Zhejiang University School of Medicine (Approval No. 20240680). Eight‐week‐old male C57/BL6J mice were randomly divided into control (CTR), lipopolysaccharide (LPS + vehicle), IN (LPS + IN), EVs (LPS + EVs), EVs@IN (LPS + EVs@IN) and FLX (LPS + FLX) groups, maintained in a stable environment (22°C–25°C) and provided with adequate water and food. The mice were sacrificed after the completion of the behaviour tests.

### 2.8 LPS‐Induced Depressed Model and Drug Administration

For the establishment of a depressed model, LPS (Sigma‐Aldrich, # L2880)dissolved in saline was injected for 14 consecutive days (1 mg/kg, i.p). Meanwhile, vehicle, IN, EVs, EVs@IN and FLX were administered in different groups, respectively, during the 8 to 14 days. Each mouse underwent adaptive training for 3 days prior to intranasal administration. The total volume is 20 µL, given in 5 portions (4 µL each time), using a micropipette with a soft tip. And the treated nostril was alternated daily to minimize irritation.

### 2.9 Chronic Unpredictable Mild Stress Procedure

The CUMS procedure was performed as previously described (Chen et al. 2024).

### 2.10 Behaviour Tests

The mice were transferred in advance for environmental adaptation to the behaviour tests. To minimize the impact of odors on the outcomes, the apparatus was disinfected with alcohol and subsequently dried following each trial. The movement pathways of the subjects were documented and a correlation analysis was performed by the animal behaviour analysis software.

For the open field test (OFT), a configuration featuring a central area of 25 cm × 25 cm was employed to assess the anxiety‐like behaviour. The subjects were promptly positioned in the centre area and permitted to move freely for 5 min. The total distance and the time spent within the centre area were recorded and analyzed by the software.

The elevated plus maze (EPM), which is situated 100 cm above the ground, comprises a central platform (6 cm × 6 cm), two enclosed arms (30 cm × 6 cm × 20 cm) and two open arms (30 cm × 6 cm), and is another test employed to assess anxiety‐like behaviours. The subjects were permitted to explore freely, and the duration spent opening the arms was recorded and analyzed.

The forced swimming test (FST) and tail suspension test (TST) were both utilized to evaluate depressive‐like behaviours. In the FST, mice were placed in a 10 cm diameter cylindrical apparatus filled with water (a depth of 30 cm, 25°C). In the TST, the tails of mice were taped in an upside‐down position, maintaining a distance of approximately 25 cm between their noses and the experimental site floor. A camera system recorded the entire 6‐min test duration and quantified the immobility time for the final 4 min, during which the mice exhibited no signs of struggle.

The behavioural tests were performed in the following order to reduce stress interference: OFT, EPM, TST and FST, with a 24‐h interval between each test.

### 2.11 Stereotactic Surgery and Virus Injection

Prior to undergoing surgery, mice were anaesthetized with 3% isoflurane and subsequently maintained under anaesthesia with 1.5% isoflurane once fixed in the stereotactic apparatus (RWD Life Sciences). A heating pad ensured that the mice maintained a constant temperature. To mark neuronal dendritic spines, AAV‐NCSP‐YFP‐2E5 (total viral titer, # BC‐SL001, BrainCase) virus was bilaterally injected into the hippocampus (100 nL per side, coordinates from bregma: AP, −2.0 mm; ML, ±1.5 mm; DV: −1.5 mm from bregma) of 7‐week‐old C56/BL6J mice to achieve sparse labelling. To establish hippocampal‐olfactory bulb‐nose multistage neural circuit, PRV‐CAG‐3Gc (8.70 × 10^9^ PFU/mL, Brain case, 300 nL for each side) was injected in bilateral hippocampus. The infusion rate was set at 1 nL/s, and the capillary needle was carefully withdrawn 10 min post‐injection. Following a 7‐day rest period after virus injection, LPS and drug administration were commenced.

### 2.12 Immunofluorescence

Mice were anaesthetized by intraperitoneal injection of sodium pentobarbital (60 mg/kg) and positioned supinely on the operating table, securely fastened to prevent any movement during the perfusion. Initially perfused with saline at a constant rate, ensuring thorough blood removal, subsequently replaced with 4% paraformaldehyde (PFA) for continued perfusion. The entire mouse brain tissue was isolated and immersed in 4% PFA (4°C) for overnight fixation, followed by dehydration in 20% and 30% sucrose solutions, respectively. The dehydrated brain tissue was embedded and sectioned into 40 µm coronal slices with a cryostat slicer system (CM1860, Leica) and stored in antifreeze solution.

Hippocampus tissue sections underwent 3 washes with PBS and then blocked in 5% BSA‐PBS at room temperature for an hour. Subsequently incubated in primary antibody (C3, 1:500, Affinity, # DF13224; S1000A10, 1:100, abcam # ab76472; GFAP, 1:700, abcam, # ab7260; Doublecortion (DCX), 1:2000, abcam, # ab18723; synaptophysin (SYP), 1:500, proteintech, # 67864‐1‐Ig; postsynaptic density protein‐95 (PSD95), 1:500, proteintech, # 20665‐1‐AP) overnight at 4°C, after rinsing with PBS, incubated with the appropriate secondary antibody at room temperature, protected from light, for another hour. Once washed with PBS again, all sections were mounted with an anti‐quenching blocker containing DAPI. To visualize the fluorescence signals, slices were imaged using a microscope (Olympus FV3000 or Leica Stellaris 5). Further quantification was performed by ImageJ.

Cells cultured on round glass coverslips were fixed with 4% PFA for 15 min at room temperature. Afterward, nonspecific binding was blocked with 2% BSA at 37°C for 30 min. Samples were then incubated overnight at 4°C with the indicated primary antibodies, followed by incubation with appropriate fluorescent secondary antibodies for 1 h at room temperature in the dark and mounted with an anti‐fluorescence quenching blocking agent.

### 2.13 Quantitative PCR (qPCR) Analysis

In summary, the total RNA of hippocampus tissue was extracted based on FastPure Cell/Tissue Total RNA Isolation Kit V2 (Vazyme, # RC112‐01) and subsequently converted into cDNA (Vazyme, # R333‐01). qPCR was carried out using the PerfectStart Green qPCR SuperMix (TransGen Biotech, #AQ601) following the procedure: 94°C, 2 min; 39 cycles: 94°C 5 s, 55°C 15 s, 72°C 10 s. The relative mRNA expressions of target genes were calculated by the 2^−ΔΔCt^ method. The detailed primer sequences are provided in Table S2.

### 2.14 RNA Sequencing (RNA‐Seq) Analysis

The entire RNA‐seq analysis was performed by Kaitai Bio. Briefly, total RNA was extracted from hippocampal tissue. Agarose gel electrophoresis was used to examine the sample for RNA integrity and the presence of DNA contamination, and preliminary quantification was performed using Nanodrop. The RNA was fragmented, end‐patched and spliced; the ligation product was purified and amplified. The 300–600 bp target product was recovered by magnetic bead purification for sequencing on an Illumina NovaSeq 6000 instrument with double‐ended 150 bp reads.

After filtering the raw data, checking the sequencing error rate, and checking the GC content distribution, we obtained clean reads for subsequent analyses, in which raw data filtering included removing reads with adapters (Weisler et al. 2016); removing reads with a ratio of N, which indicates the base information could not be determined, greater than 0.002 (Ding et al. 2016); removing the paired reads where the number of low‐quality bases in the single‐ended read exceeds 50% of the length of the read. Hisat2 software was used to compare the resulting validated sequencing data with the Ensembl database annotation using default parameters. Subsequently, the RNA‐Seq data were spliced and searched for transcripts using StringTie. Quantitative analysis was performed using the union mode of the HTSeq software, and gene expression values were expressed as RPKM or FPKM. Once the quantitative analysis was completed and the expression matrix of all samples was obtained, expression difference significance analysis could be performed at the gene or transcript level to find functional genes or transcripts of interest. The expression difference significance analysis was performed using edgeR software, with padj < 0.05 as the significance criterion for difference. The clusterProfiler software was used for pathway enrichment analysis of differential genes.

### 2.15 Transmission Electron Microscopy (TEM) Analysis

Hippocampal tissue samples were collected and immediately immersed in a 2.5% glutaraldehyde solution for overnight fixation. The fixed tissue samples were transferred to 1% osmium tetroxide solution for further fixation at room temperature for 2 h. The samples were thoroughly rinsed three times with PBS, then dehydrated in a series of increasing concentrations of ethanol solutions (50%–100% and 100%, respectively), each stage lasting 15 min and followed by 100% acetone (twice, 20 min per time), embedded in Epon and hardened to produce ultrathin sections stained with lead citrate before ultrastructural images of hippocampal synapses were captured by transmission electron microscopy (Tecnai G2 spirit 120 kV).

### 2.16 Lactic Acid and Oxidative Stress‐Related Indicators Measurement

The concentrations of lactic acid (#A019‐2‐1, Nanjing Jiancheng Bioengineering Institute) and oxidative stress indicators (including glutathione [GSH], superoxide dismutase [SOD] and malondialdehyde [MDA]) were assayed in accordance with the manufacturer's guidelines (Reduced GSH Content Assay Kit, Solarbio, # BC1175; SOD Activity Assay Kit, Solarbio, # BC5165; MDA Content Assay Kit, Solarbio, # BC0025). In summary, hippocampus tissue was mixed with PBS in a 1:9 volume ratio, homogenized while kept in an ice bath, followed by centrifugation at 12000 rpm for 10 min, and the supernatant was isolated for further quantification.

### 2.17 Cells Cultures

BV2 microglial cells and HT22 hippocampal neuronal cells were cultured in high‐glucose Dulbecco’s Modified Eagle Medium (DMEM, BDBio, # L100‐500) supplemented with 10% (v/v) fetal bovine serum (FBS, BDBio, # F801‐500) and 1% penicillin‐streptomycin (Gibco, # 15140122). For long‐term storage, cells in the logarithmic growth phase were harvested, resuspended in CELLSAVING (#C40050, NCM biotech) and stored at −80°C. Prior to experimental treatments, cells were seeded onto appropriate culture plates or glass coverslips. Before collecting the BV2 cell culture medium (MCM), expose BV2 to the following culture medium for 24 hours: (I) Equal amounts of PBS; (II) LPS (100ng/mL).

Primary astrocyte cultures were established from hippocampi of neonatal (postnatal day 0‐1) C57BL/6J mice under sterile conditions. Dissected tissues were enzymatically dissociated using 0.25% trypsin for 15 minutes at 37°C, followed by gentle trituration to obtain a single‐cell suspension. Cells were seeded into poly‐D‐lysine–coated (PDL; Thermo Fisher, #A3890401) T75 culture flasks and maintained in DMEM/F‐12 medium (BDBio, #L104‐500) containing 15% fetal bovine serum (BDBio, #F801‐500) and 1% penicillin‐streptomycin. Cultures were incubated at 37°C with 5% CO_2_, and the medium was replaced every two days. After 10‐12 days of growth, astrocytes were purified by orbital shaking at 200 rpm for 12 h. Before collecting the astrocyte culture medium (ACM), expose astrocytes to the following culture medium for 24 hours: (I) Equal amounts of PBS; (II) MCM(LPS).

### 2.18 CCK‐8 Assay

Cell viability was determined using the Cell Counting Kit‐8 following the manufacturer’s protocol. Briefly, astrocytes and HT22 cells were plated in 96‐well plates. Following adherence, cells were then exposed to varying concentrations of EVs (0, 2.5, 5, 10, 20, 40 µg/mL) for 24 h. After treatment, 10 µL of CCK‐8 reagent was added to each well containing 100 µL of medium, and the plates were incubated for 1–2 h at 37°C. Absorbance at 450 nm was recorded using a microplate reader (BioTek), and cell viability was expressed relative to the untreated control.

### 2.19 Mitochondrial Stress Test

The oxygen consumption rate (OCR) was determined using an XFe24 analyzer (Agilent) with the Seahorse XF Cell Mito Stress Test Kit (Agilent, # 103015‐100). Astrocytes (8 × 103 cells/well) and HT22 neuronal cells (1 × 10⁴ cells/well) were plated in 24‐well Seahorse XF microtiter plates and maintained overnight at 37°C in a humidified 5% CO_2_ atmosphere. Cells were exposed to different conditioned media treatments, including PBS, MCM(LPS), MCM(LPS)+EVs (5 µg/mL) for astrocytes, and PBS, ACM(LPS), ACM(LPS)+EVs (5 µg/mL) for HT22 cells. Before assay measurements, the culture medium was exchanged for Seahorse XF assay medium (#103575‐100) supplemented with 10 mM glucose (#103577‐100), 2 mM glutamine (#103579‐100), and 1 mM sodium pyruvate (#103578‐100), followed by a 1 h equilibration period in a CO2‐free incubator at 37°C. During the assay, oligomycin (1 µM), FCCP (1 µM), and rotenone and antimycin A (0.5 µM) were sequentially injected through the sensor cartridge ports. Data acquisition and calculation of respiratory parameters were performed using Agilent Wave software.

### 2.20 Statistical Analysis

The number of experimental repetitions (*n*) is specified in the legend and corresponds to the number of independently treated experimental subjects per condition. Statistical methods were not employed to pre‐determine sample size or randomize assignments. Normality was assessed using the Shapiro–Wilk test for all datasets. For normally distributed data, comparisons between two groups were conducted using the Student's *t*‐test, while ANOVA with Dunnett's post‐hoc test was applied for comparisons involving more than two groups. Non‐parametric tests were utilized for non‐normally distributed data: the Mann–Whitney *U* test for pairwise comparisons, the Kruskal–Wallis test for comparisons involving more than two groups. Statistical analyses were performed using Prism (v7.0). The significance threshold was set at *p* ≤ 0.05, with levels denoted as *p ≤ 0.05, **p ≤ 0.01 and ***p ≤ 0.001.

## 3 Results

### 3.1 Fabrication and Structural Characterization of EVs@IN

**FIGURE 1:**
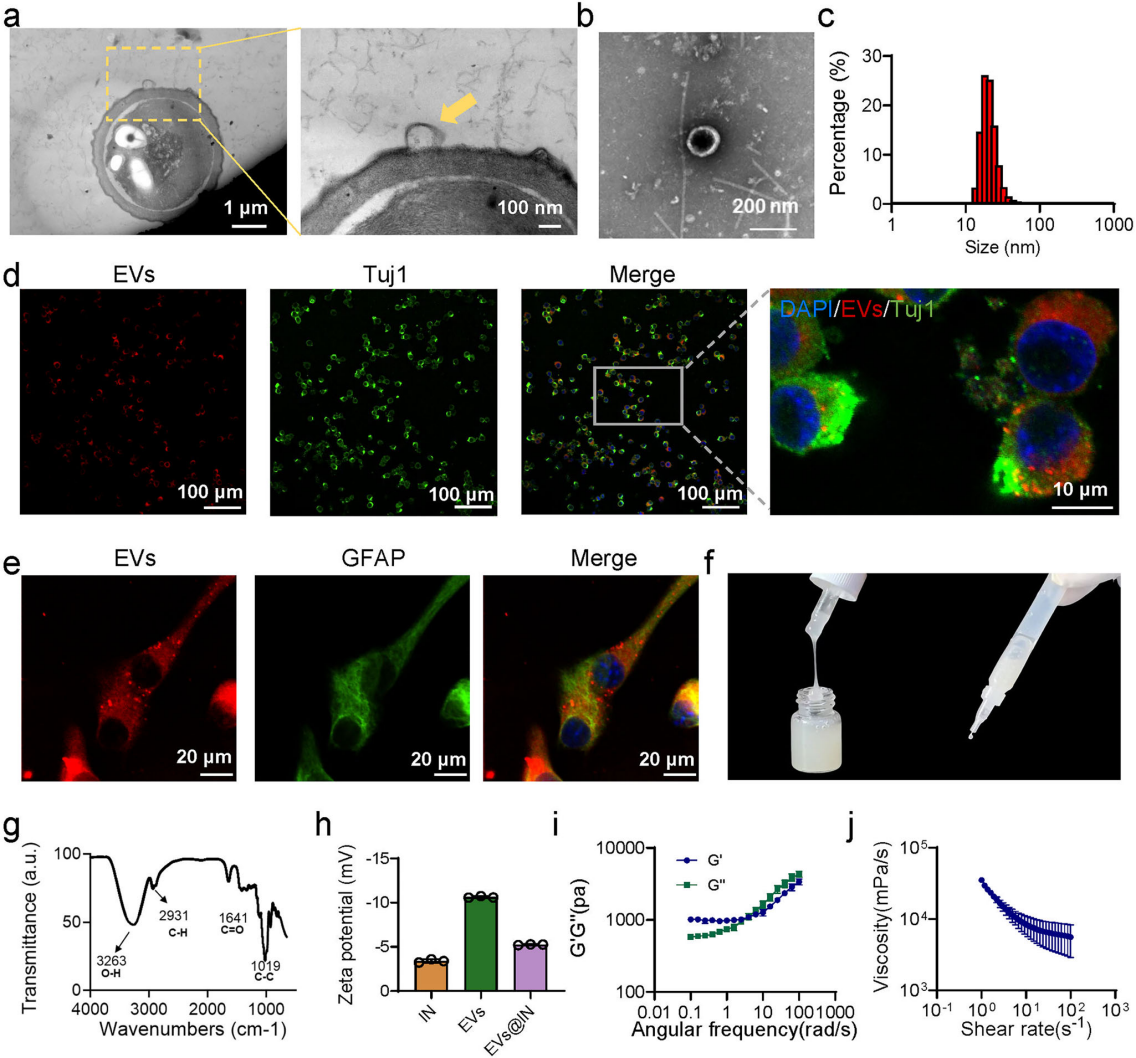
Fabrication and structural characterization of EVs@IN. (a) TEM image of EVs release (yellow arrow: EVs on CV; inset: EVs). Scale bars, 1 µm and 100 nm, respectively. (b) High‐resolution TEM of EVs with lipid bilayer. Scale bar, 200 nm. (c) DLS analysis of EVs. (d and e) EVs were taken up and utilized by HT22 (d) and astrocytes (e) after co‐culture with them, respectively. Scale bars, 100, 10 and 20 µm, respectively. (f) Morphological representation of the as‐prepared EVs@IN. (g) FTIR spectra of EVs@IN. (h) Zeta potential of IN, EVs and EVs@IN. (i) Frequency‐dependent rheology of EVs@IN. (j) Shear‐thinning viscosity profile of EVs@IN.

*C. vulgaris* is a sustainable source of EVs (Figure S1a). SEM shows CV cells are spherical, smooth and 2–5 µm in diameter (Figure S1b). Optical microscopy confirms this structure, and fluorescence microscopy reveals chlorophyll‐derived autofluorescence (Figure S1c,d). CV can secrete EVs dynamically, while TEM provided direct evidence of biogenesis by capturing vesicle budding from the CV membrane (orange arrowhead, Figure 1a). High‐resolution imaging further revealed intact, bilayered EVs with characteristic cup‐ or sphere‐like morphology (Figure 1b). DLS analysis showed a monodisperse population centred at 93.97 nm (PDI 0.229), consistent with typical EV sizes (Figure 1c). We confirmed in vitro that it can be swallowed by astrocytes and neuronal cells (Figure 1d,e) and is safe against them (Figure S1e). We used inulin, a common inhaled vaccine adjuvant, to assemble a gel to address the short retention time of the nasal cavity. First, the IN hydrogel (IN) was prepared by dissolving inulin, followed by incorporation of EVs via ultrasonication to form EVs@IN (Figure S1f). Gelation of inulin was not observed in the absence of heating (Figure S1g). The transition from free‐flowing liquid (EVs) to non‐flowing viscous gel (IN) and semi‐flowable EVs@IN (retaining partial shape upon tube inversion) suggests successful physical incorporation of EVs into the IN‐based gel matrix (Figures 1f, S1h). FTIR analysis of EVs@IN revealed characteristic peaks corresponding to inulin (Figure 1g), indicating structural preservation of the gel matrix during encapsulation. The zeta potential analysis differentiated surface charges across formulations: Compared to IN and EVs, EVs@IN showed a moderate zeta potential, suggesting partial surface masking while retaining negative charge (Figure 1h). EVs@IN exhibited optimal nasal delivery properties: Elastic dominance (G' > G″ across 10^1^–10^3^ rad/s, Figure 1i) ensures prolonged mucosal retention, while shear‐thinning behaviour (Figure 1j) enables easy dropwise instillation—resolving the conflict between durability and administrability in nasal vaccines. Furthermore, the hydrogel retained its structural integrity and remained readily injectable for over 7 days when stored at 4°C (Figure S2a), supporting its viability for clinical administration. Beyond the stability of the formulated hydrogel, the consistent production of the EVs' building blocks is equally critical for translational applications. To rigorously assess the reproducibility of our isolation protocol, we performed three independent preparations. The results demonstrated consistent batch‐to‐batch characteristics, with an average particle size of 91.73 ± 2.05 nm, a polydispersity index (PDI) of 0.201 ± 0.028 and a total protein yield of 12.87 ± 1.26 mg (mean ± SD, *n* = 3; Figure S2b), underscoring the robustness of our EV production process. In addition, EVs@IN present temperature‐dependent release kinetics of EVs from IN in PBS (Figure S3a–c), highlighting hydrogel responsiveness to physiological conditions.

### 3.2 Biodistribution and Biodegradation of EVs@IN

**FIGURE 2:**
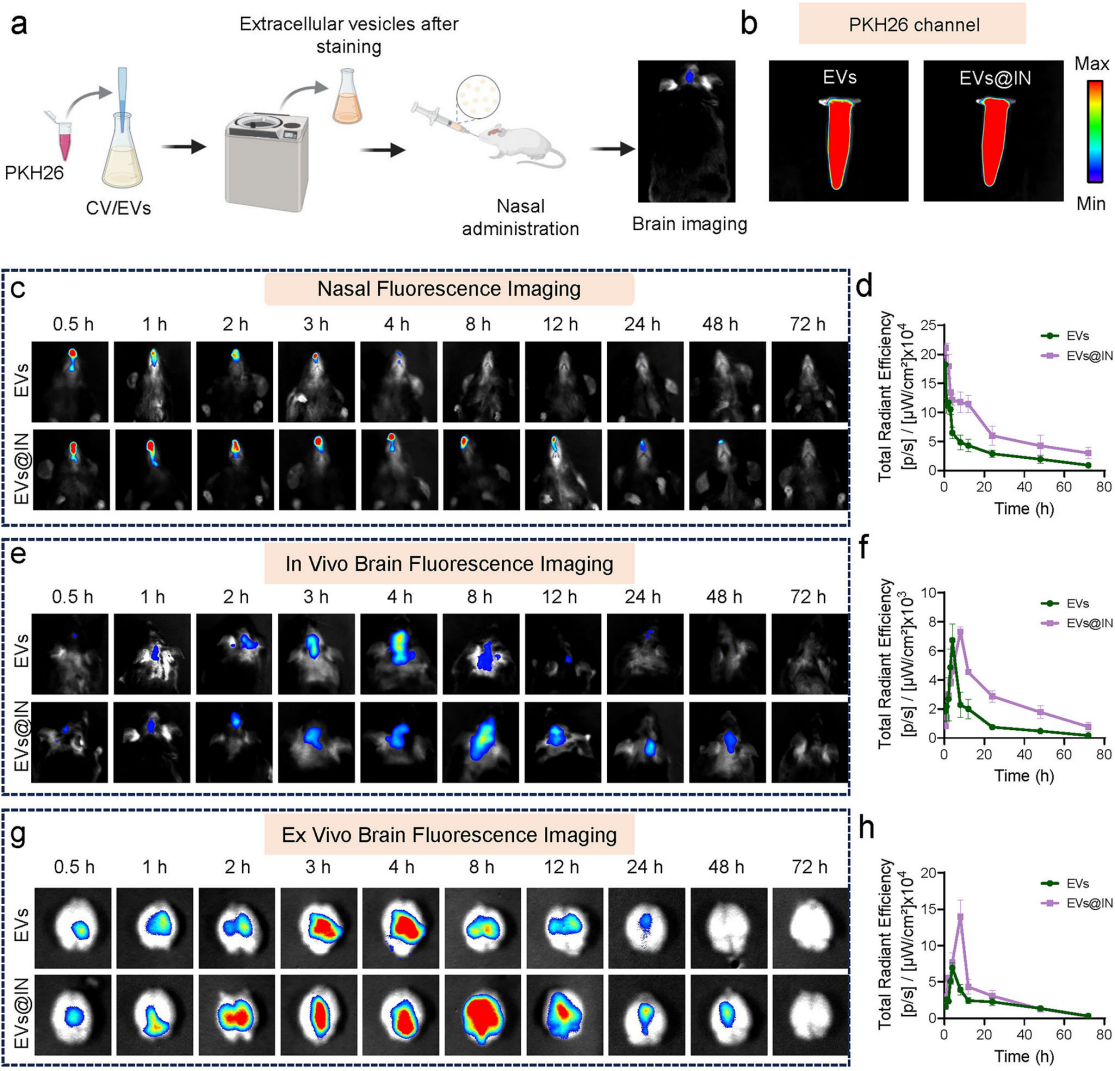
Biodistribution and retention of EVs@IN following intranasal administration. (a) Schematic illustration of intranasal administration of EVs and EVs@IN. (b) Fluorescence imaging of PKH26‐labelled EVs and EVs@IN. (c) Nasal cavity fluorescence imaging of mice at different time points post EVs/EVs@IN administration (PKH26 channel). (d) Quantification of nasal cavity fluorescence signals over time (EVs@IN vs. EVs). (e) In vivo brain fluorescence imaging of mice at different time points posts EVs/EVs@IN administration. (f) Quantification of brain fluorescence signals over time (EVs@IN vs. EVs). (g) Ex vivo brain fluorescence imaging of mice post EVs/EVs@IN administration. (h) Quantification of ex vivo brain fluorescence signals over time (EVs@IN vs. EVs).

To visualize the in vivo distribution of EVs, fluorescent labelling was performed using PKH26 dye. Labelled‐EVs were administered nasally to mice, and their distribution was monitored using a small animal imaging system (Figure 2a). Fluorescence imaging confirmed the properties of both EVs and EVs@IN, with excitation at 551 nm and emission at 567 nm (Figure 2b). Nasal retention was evaluated by intranasal administration of EVs and EVs@IN to C57BL/6J mice, with fluorescence signals monitored at various time points (0.5‐72 h). EVs@IN showed stronger and longer‐lasting signals in the nasal region compared to EVs, indicating improved retention (Figure 2c,d). In vivo brain imaging revealed that EVs accumulated gradually, peaking at 4 h and fading by 24 h, while EVs@IN reached higher intensity at 8 h and maintained detectable signals up to 48 h, suggesting enhanced brain distribution and retention (Figure 2e,f). Ex vivo brain imaging further supported these findings, confirming sustained release and retention of EVs@IN (Figure 2g,h).

Ex vivo imaging of major organs explored the distribution patterns of the formulations. Intranasal EVs caused fluid‐induced irritation, leading to choking and lung fluorescence, increasing the risk of aspiration pneumonia. In contrast, EVs@IN, due to its higher viscosity, avoided such complications. A small amount of drug entered the gastrointestinal tract (Figure S3d), and faecal fluorescence imaging showed efficient breakdown, metabolism and excretion via the GI tract, demonstrating safety (Figure S3e).

To exclude interference from free dye, control experiments with PKH26 and PKH26@IN were conducted. Neither control group showed significant fluorescence in the nose, brain or major organs (Figure S4), confirming that the observed biodistribution was specific to EVs.

### 3.3 Transport of EVs From the Nose to the Hippocampus

**FIGURE 3:**
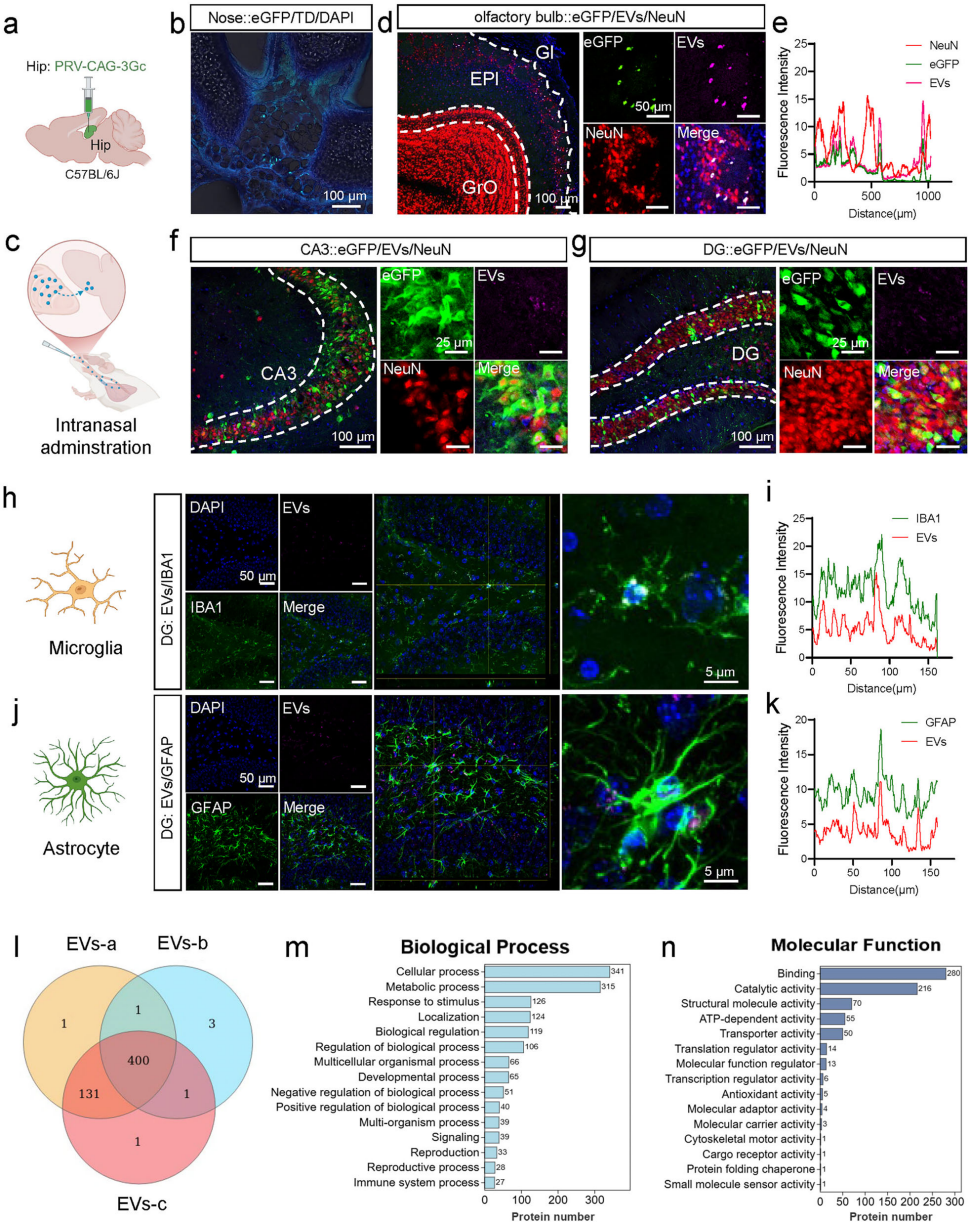
EVs can directly enter the brain and act on different glial cells or neurons by nasal delivery. (a) The strategy of virus injection. PRV‐CAG‐3Gc injected into bilateral hippocampus. (b) Immunofluorescence images of eGFP expression in the brain. Scale bar, 100 µm. (c) Pattern diagram of intranasal administration. (d and e) Immunofluorescence images of eGFP, EVs and NeuN in the olfactory bulb (d) and the analysis of co‐localization (e). Scale bars, 100 and 50 µm, respectively. (f and g) Immunofluorescence images of eGFP, EVs and NeuN in CA3 (f) and DG (g) in the hippocampus. Scale bars, 100 and 25 µm, respectively. (h and i) Immunofluorescence images (h) and the analysis of co‐localization (i) of EVs and IBA1 in the hippocampus. Scale bars, 50 and 5 µm, respectively. (j and k) Immunofluorescence images (j) and the analysis of co‐localization (k) of EVs and GFAP in the hippocampus. (l) Sample association analysis of EVs of *Chlorella vulgaris* from different batches. (m and n) Gene Ontology analysis of biological processes (m) and molecular function (n) of EVs proteins.

Olfactory nerves act as a key link between the nasal cavity and the central nervous system, forming olfactory pathways whose axons penetrate the sieve plate that separates the nose from the brain. The hippocampus, the brain's emotional centre, is closely related to depression. We first injected PRV into the hippocampus and confirmed the existence of a hippocampal‐olfactory bulb‐nose multistage neural circuit (Figure 3a,b). We then labelled EVs with PKH26 as a tracer molecule to track the transport of potential therapeutic payloads from the nose to the hippocampus, and we found that EVs were transported along neural circuits and distributed in multiple subregions of the hippocampus (Figure 3c–g). To explore the possible modes of EVs transport, we further determined the presence of EVs in the brain. By immunofluorescence staining with microglia, astrocytes and neuronal markers IBA1, GFAP and NeuN, respectively, we found that EVs co‐labelled with all three, indicating that different kinds of cells have the potential to utilize EVs (Figure 3c–k). Therefore, EVs may be able to directly enter the brain and act on different glial cells or neurones by nasal delivery.

### 3.4 EVs@IN Relieves LPS‐Induced Depressive and Anxiety‐Like Behaviours

We further extracted EV proteins to analyze the possible functional role of EVs. Sample association analysis showed that EVs from different batches had good consistency (Figure S5a), suggesting that EVs from Chlorella had the potential to expand production, among which 400 proteins were present in all three batches of samples (Figure 3l). Gene Ontology analysis demonstrated the function of EV proteins at three levels. In terms of biological processes, it may be involved in metabolic processes, response to stimulus and immune system processes (Figure 3m). In terms of molecular function, it may regulate ATP‐dependent activities as well as antioxidant activities (Figure 3n). KEGG pathway analysis was used to determine the role of proteins in EVs in metabolic signalling pathways (Figure S5b). Thus, EVs may play a role in energy metabolism regulation.

**FIGURE 4:**
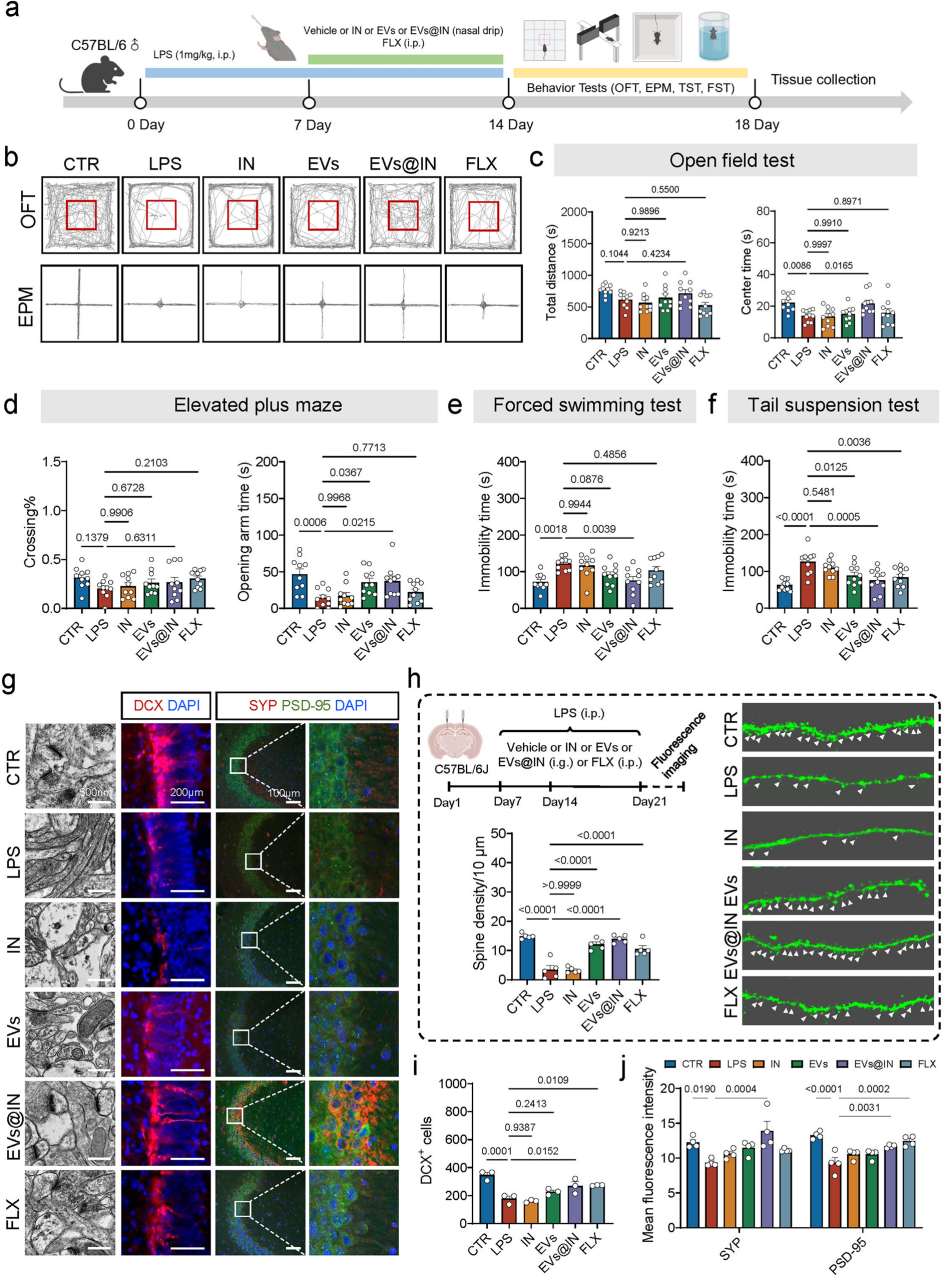
EVs@IN relieves LPS‐induced depressive and anxiety‐like behaviours. (a) Schematic of experimental design. After establishing LPS‐induced depression model and combining treatment with Vehicle, Inulin (IN), EVs, EVs@IN and fluoxetine (FLX), we sequentially performed open field test (OFT), elevated plus maze (EPM), forced swimming test (FST) and tail suspension test (TST) (*n* = 10). (b) Typical path map in the OFT and EPM in different groups. (c) The total distance and centre time in OFT in different groups. (d) The percentage of crossings and time in opening arms in different groups. (e and f) The immobility time in FST (e) and TST (f) in different groups, respectively. (g) Representative transmission electron microscopy and immunofluorescence images in hippocampus in different groups. (h) Schematic of experimental design and the representative images and quantifications of hippocampal dendritic spines (*n* = 5). (i) The quantification of the number of doublecortin (DCX) positive cells in DG (*n* = 3). (j) The quantification of the mean fluorescence intensity of synaptophysin (SYP) and postsynaptic density protein‐95 (PSD‐95) (*n* = 4). Data are represented as means ± SEM. The significance of difference in (c–f) and (h–j) was determined by one‐way ANOVA with Dunnett's post‐hoc test.

We previously found that depression is linked to hippocampal energy metabolism disruption and neuroinflammation. To investigate the efficacy of EVs and EVs@IN, we established a depressive‐like model using a 7‐day LPS injection (Yin et al. 2023). Fluoxetine (FLX), a widely used antidepressant, served as the positive control. During continued LPS injection, IN, EVs and EVs@IN were administered intranasally for 7 days, while FLX was given intraperitoneally (Figure 4a). Behavioural tests assessed changes in depressive and anxiety‐like behaviours: open field test (OFT) and elevated plus maze (EPM) for anxiety and tail suspension test (TST) and forced swimming test (FST) for depression. Results showed that LPS‐treated mice spent significantly less time in the centre area during OFT. In contrast, EVs@IN treatment increased this time (Figure 4b,c). Similarly, LPS‐induced reductions in open‐arm time in the EPM were rescued by both EVs and EVs@IN treatments (Figure 4d), indicating EVs@IN reverses anxiety‐like behaviours. In the FST and TST, EVs partially decrease LPS‐induced immobility time, while EVs@IN show greater improvement (Figure 4e,f). These findings suggest EVs@IN effectively alleviates depressive and anxiety‐like behaviours.

### 3.5 EVs Involved in Regulating Neuroplasticity and the Critical Functional Complement System

Synaptic damage and neurogenesis disruption in the hippocampus have been suggested as potential mechanisms for the development of depression (Sheline et al. 2002; Nestler et al. 2002; Tartt et al. 2022; Duman and Aghajanian 2012). Interestingly, EVs@IN treatment could significantly ameliorate the LPS‐induced marked reduction in the number of Doublecortin^+^ (DCX) cells, an indicator of adult neurogenesis (Li et al. 2021) (Figure 4g,i). Meanwhile, to observe alterations in neuronal dendritic spines, we injected sparsely labelled virus into the hippocampus of mice with different interventions (Figure 4h). Dendritic spines are the main site of synapse formation between neurones, and a decrease in their number affects synaptic plasticity (Sala and Segal 2014). Correspondingly, neuronal dendritic spines in the hippocampus of the LPS‐treated group were significantly reduced and significantly improved after EVs, EVs@IN and FLX treatments (Figure 4h). We also evaluated the impact on synaptic plasticity in the hippocampus, in which results consistently showed that the expression of the synapse‐related proteins (SYP and PSD‐95) was significantly diminished in the LPS‐treated group and could be significantly improved by EVs@IN treatment (Figure 4g,j). Furthermore, transmission electron microscopy (TEM) of hippocampal tissue showed that LPS‐induced damage to the dense postsynaptic region was also attenuated in the EVs@IN group (Figure 4g).

**FIGURE 5:**
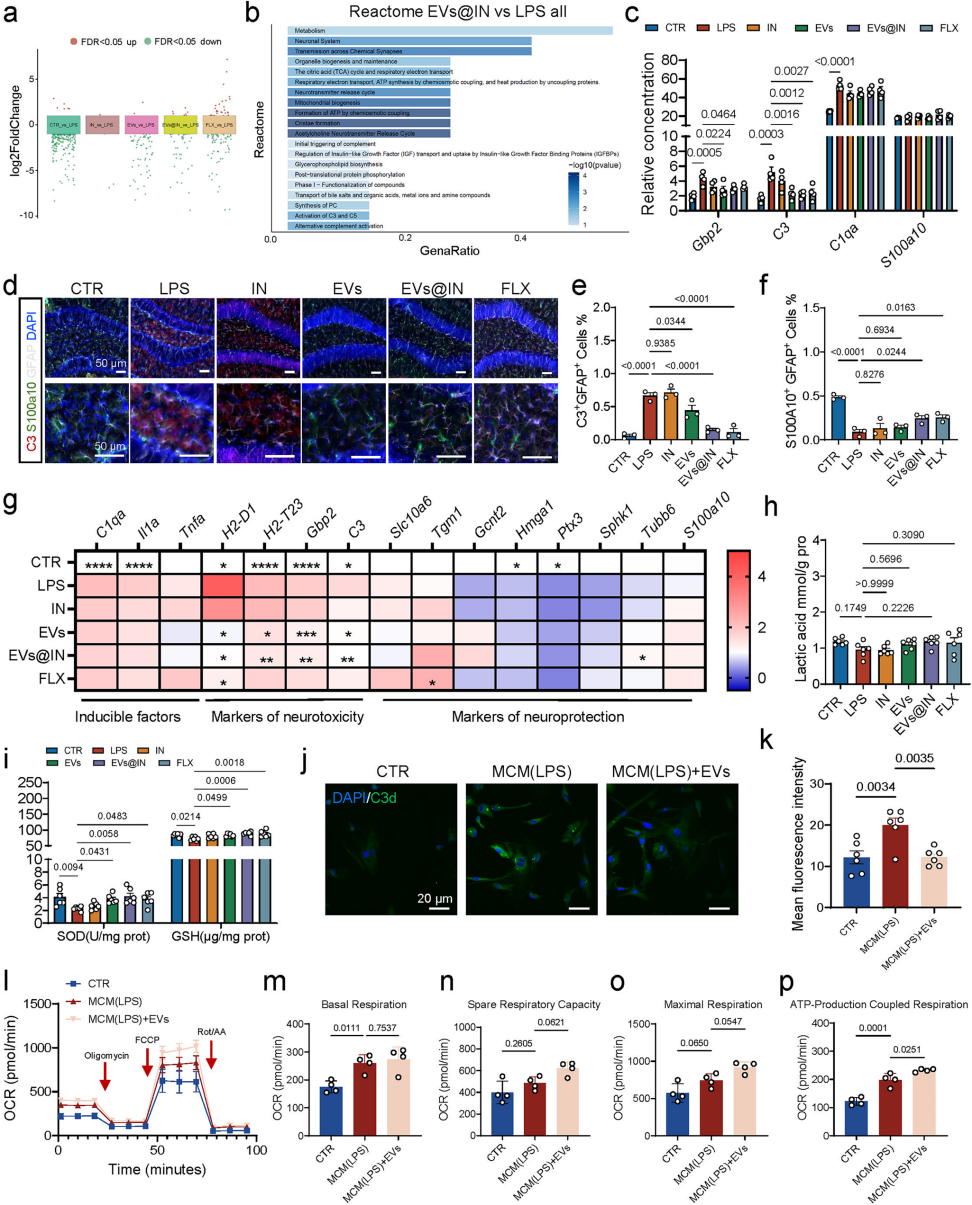
EVs@IN involved in regulating astrocyte subtypes and the critical functional complement system. (a) The gene expression changes in hippocampus in different groups. (b) Reactome enrichment analysis between EVs@IN and LPS groups. (c) The relative expression of neurotoxic and neuroprotective astrocytes markers in hippocampus (*n* = 4). (d–f) Representative images (d) and the quantification of the percentage of C3^+^GFAP^+^ cells (e) and S100A10^+^GFAP^+^ cells (f) in hippocampus (*n* = 3). (g) The mRNA expression of neurotoxic astrocytes inducer, neurotoxic and neuroprotective astrocytes markers in hippocampus in different groups (*n *= 6). (h) The lactic acid level in hippocampus in different groups (*n* = 6). (i) The activity of superoxide dismutase (SOD) and the level of glutathione (GSH) in hippocampus in different groups (*n* = 6). (j–k) The representative images (j) and the quantification of mean fluorescence intensity of C3d (k) (*n* = 6). (l–p) The results of astrocyte Cell Mito Stress Test in different treatment, including oxygen consumption rate (OCR) (l), basal respiration (m), spare respiratory capacity (n), maximal respiration (o) and ATP‐production coupled respiration (p) (*n* = 4). Data are represented as means ± SEM. The significance of difference in (c), (e–i), (k) and (m–p) was determined by one‐way ANOVA with Dunnett's post‐hoc test while the significance of difference in the expression of *S100a10* in (c) and the expression of *Tgm1* and *Hmga1* in (g) was determined by Kruskal–Wallis test. *

To further explore the mechanisms by which EVs@IN alleviate depressive and anxiety‐like behaviours, we performed RNA sequencing (RNA‐Seq) on hippocampal tissue collected immediately after behavioural tests. RNA‐Seq results showed that the EVs@IN‐treated group exhibited significant gene expression changes compared to the LPS‐treated group, including 7 upregulated genes and 34 downregulated genes (log2FC > 1, padj < 0.05, Figures 5a and S6a). Reactome analysis revealed differences in three main pathways: synaptic function, energy metabolism and complement activation, particularly involving complements C3 and C5 (Figure 5b). Abnormal complement activation, especially through complement C3, is a critical factor in neurological disorders, as C3 is central to the complement system and plays a role in neurodevelopment and neurotoxicity (Chen et al. 2024). Notably, complement C3, a key neurotoxic astrocyte marker, was significantly downregulated (Figure 5c). Astrocytes are the primary source of complement C3 and are linked to central energy metabolism and synaptic plasticity (Hammond et al. 2020). Additionally, C3 is highly upregulated in neurotoxic astrocytes (Liddelow et al. 2017). Treatment with EVs and EVs@IN significantly reduced the mRNA expression of neurotoxic astrocyte markers *Gbp2* and *C3*, while the inducer *C1qa* and the neuroprotective marker *S100a10* remained unchanged (Figure 5c). In summary, EVs@IN may specifically inhibit neurotoxic reactive astrocytes in the hippocampus, thereby alleviating depressive and anxiety‐like behaviours.

### 3.6 The Regulatory Role of EVs@IN on Astrocyte Subtypes

We further explored the regulatory effects of EVs@IN on astrocyte subtypes. Immunofluorescence analysis of the hippocampus was conducted; the results demonstrated that LPS treatment significantly increased the percentage of C3^+^GFAP^+^ cells (Figure 5d,e), indicating a marked proliferation of neurotoxic astrocytes. This finding from qPCR data revealed significant upregulation of mRNA expressions for other A1 reactive astrocyte markers, including *H2‐D1*, *H2‐T23* and *Gbp2*, in the LPS‐treated group (Figure 5g). However, these effects were substantially mitigated by EVs@IN treatment (Figure 5d,e,g). Additionally, EVs@IN treatment significantly elevated the mRNA expression levels of certain neuroprotective astrocyte markers (Figure 5g). Immunofluorescence results also showed that EVs@IN treatment could reverse the LPS‐induced reduction in the percentage of S100A10^+^GFAP^+^ cells (Figure 5d,f). Moreover, experiments using primary astrocytes confirmed that EVs effectively inhibited the generation of C3^+^ astrocytes (Figures 5j,k and S7a,c), providing further evidence of EVs' role in regulating astrocyte subtypes.

It is well known that cytokines (IL‐1α, TNF‐α and C1Qa) secreted by microglia induce neurotoxic astrocytes. However, no significant changes in these inducers were found after treatments (Figure 5g). Although a significant increase in IBA1^+^ cells and a significant change in microglial morphology, specifically a larger nucleus and shorter branch length, were observed in the LPS‐treated group, no improvement was noted in the different treatment groups (Figure S7d, f). At the same time, microglial phagocytosis activity measured by CD68, a marker that distinguishes resting and activated microglia (Xu et al. 2023), was detected. Despite a significant increase in the proportion of CD68^+^ microglia in the LPS‐treated group, no improvement was observed in the treatment groups (Figure S7e,f). It indicates that EVs@IN could directly regulate astrocyte subtypes rather than microglia.

### 3.7 EVs@IN Participates in Oxidative Stress Processes and Regulating Astrocyte Phenotypic Transformation

**FIGURE 6:**
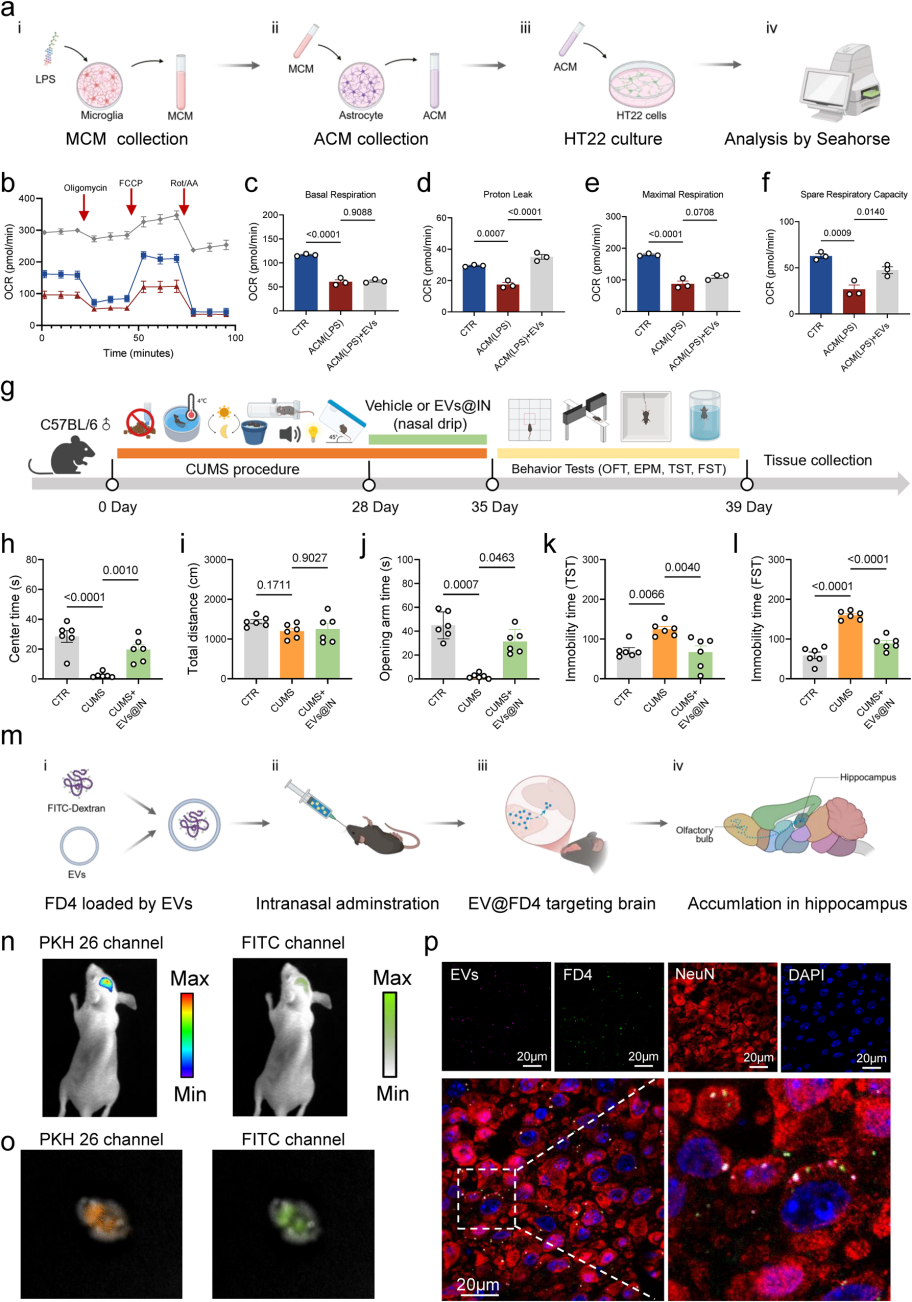
EVs can be used as a carrier for naso‐brain delivery. (a) Schematic of the experimental design. LPS stimulated microglia for 6 h, the culture medium (microglia medium, MCM) was collected for addition to astrocytes and cultured for 24 h, the medium (astrocyte medium, ACM) was collected and co‐cultured with HT22 cells for 24 h to perform the Cell Mito Stress Test. (b–f) The results of astrocyte Cell Mito Stress Test in different treatment, including oxygen consumption rate (OCR) (b), basal respiration (c), proton leak (d), maximal respiration (e) and spare respiratory capacity (f) (*n* = 3). (g) Schematic of experimental design. (h–i) The total distance and centre time in OFT in different groups. (j) The time in opening arms in different groups. (k–l) The immobility time in TST and FST in different groups. (m) Schematic of the experimental design. (n–p) In vivo (n) and ex vivo (o) fluorescence imaging of mice following intranasal administration of PKH26‐labelled EVs@FD4. (p) Immunofluorescence images of EVs, FD4, NeuN and DAPI in CA3 of the hippocampus. Scale bars, 20 µm. Data are represented as means ± SEM. The significance of difference in (c–f), (h and i) and (k and l) was determined by one‐way ANOVA with Dunnett's post‐hoc test, while the difference in (j) was determined by Kruskal–Wallis test.

The transformation of neurotoxic astrocyte subtypes may lead to neurological dysfunction via two pathways: disrupted neural support and activated neurotoxic signals (Kalinichenko et al. 2023; Shi et al. 2022). Chronic LPS exposure induces neurotoxic astrocyte proliferation in the mouse hippocampus, damaging synapses and neurones through complement pathway activation and pro‐inflammatory factor release (Li et al. 2021; Li et al. 2022). Astrocyte‐derived lactate fuels neurones (Zhang et al. 2024), our recent studies link lactate levels to depression (Chen et al. 2024). However, no significant differences were observed in hippocampal lactate levels between the LPS group and other groups (Figure 6e). We also examined superoxide dismutase (SOD) activity and glutathione (GSH) levels, finding both significantly reduced in LPS‐treated mice but alleviated by EVs, especially EVs@IN (Figure 5i). No differences were observed in myeloperoxidase (MPO) activity between groups (Figure S7g). To explore oxidative stress reduction mechanisms, we monitored related gene expression. Peroxisome proliferator activated receptor γ coactivator‐1α (PGC‐1α), a key regulator of mitochondrial biogenesis and oxidative metabolism (Lin et al. 2005), activates nuclear factor erythroid 2‐related factor 2 (Nrf2) to reduce reactive oxidant production and protect against nerve damage (Wu et al. 1999). qPCR results showed decreased mRNA expression of *Ppargc1a* and *Nfe2l2* in LPS‐treated mice, reversed by EVs@IN treatment (Figure S7h,i), suggesting EVs@IN regulates oxidative stress via the PGC‐1α‐Nrf2 pathway. In vitro, EVs enhanced ATP‐production coupled respiration in astrocytes (Figure 5l–p) and spare respiration in HT22 cells(Figure 6a–f).

### 3.8 EVs@INAlleviates Anxiety and Depression‐Like Behaviours in Chronic Unpredictable Mild Stress (CUMS) Models

To further verify the antidepressant effect of EVs@IN, we developed a CUMS model. Consistent with the previous results, intranasal administration of EVs@IN for 1 week reversed CUMS‐induced anxiety and depression‐like behaviours (Figure 6g–l).

### 3.9 EVs Has the Ability to Deliver Drugs as a Carrier

Beyond their therapeutic potential, CV‐derived EVs are efficient drug carriers. FITC‐dextran (FD4) was loaded into EVs using passive incubation and sonication (EVs@FD4). Although no visible colour change was observed with increasing FD4 doses (Figure S8a), quantitative analysis demonstrated that sonication significantly enhanced encapsulation efficiency compared to passive loading (26.5% ± 2.4% vs. 22.95% ± 1.2%) at the maximum initial drug feeding amount of 4.8 mg (Figure S8b). There was a visual difference in colour between native EVs, FD4 and EVs@FD4 (Figure S8c). Dynamic light scattering (DLS) showed a Z‐average size of 213.8 nm and PDI of 0.425, indicating increased particle size and broader size distribution compared to unloaded EVs (93.97 nm) (Figure S8d). Fluorescence spectra analysis revealed that EVs@FD4 demonstrated characteristic fluorescence emissions of both FD4 and EVs within the 450–550 nm (Figure S8f). Zeta potential measurements showed EVs@FD4 were intermediate between EVs and FD4 (Figure S8g). Fluorescence imaging of intranasally administered PKH26‐labelled EVs@FD4 revealed detectable signals in both in vivo and ex vivo brain tissues, supporting their potential for targeted brain delivery (Figure 6m–p).

### 3.10 Biosafety Assessment

Therapeutic efficacy and biological safety are prerequisites for clinical drug translation. We evaluated the nasal drop biocompatibility of PBS, EVs, IN and EVs@IN in male C57BL/6J mice over one month. Blood routine and biochemical analyses were performed on whole blood and serum, while major organs (brain, heart, liver, spleen, lung, kidney, stomach and intestine) underwent histopathological assessment (Figure S9). Results showed no significant differences in blood parameters or organ pathology between treatment and control groups, with all indicators within normal ranges (Figure S9a–c). These findings indicate that EVs@IN and its components exhibit excellent long‐term biocompatibility without haematotoxicity or organ toxicity.

### 3.11 Discussion and Conclusion

The development of effective and safe therapeutic strategies for major depressive disorder remains a critical challenge, particularly given the limitations of current antidepressants in terms of delayed efficacy, systemic side effects and low remission rates (Marwaha et al. 2023; Page et al. 2024). In this study, we developed a novel intranasal delivery system, EVs@IN, which combines Chlorella‐derived extracellular vesicles (CV/EVs) with an inulin‐based hydrogel. Our findings demonstrate that EVs@IN effectively alleviate LPS‐induced and CUMS‐induced depressive and anxiety‐like behaviours in mice by modulating astrocyte phenotypes, reducing activation of the complement pathway and oxidative stress and restoring synaptic plasticity. These results highlight the potential of plant‐derived EVs as a promising therapeutic platform for brain‐targeted delivery.

EVs@IN demonstrated sustained release kinetics both in vivo and in vitro, ensuring prolonged therapeutic exposure. In vivo fluorescence imaging revealed enhanced nasal retention and brain accumulation for EVs@IN compared to free EVs, likely due to the adhesive properties of inulin gel. Additionally, we confirmed the presence of a multistage neural circuit from the nasal cavity to the olfactory bulb and hippocampus, demonstrating that EVs are transported along this pathway and taken up by various cells in the hippocampus. The slow‐release effect in the nasal cavity facilitates efficient transport via the olfactory pathway, bypassing the blood–brain barrier while minimizing systemic exposure. Notably, EVs@IN also avoided lung accumulation observed with free EVs, potentially reducing side effects such as coughing and pneumonia.

Our proteomic analysis of EVs suggests that their role may be related to glial cells and central energy metabolism. In fact, glial cells play a role in maintaining the physiological and pathological functions of the central nervous system (Lu et al. 2025). Astrocytes in particular are important contributors to the pathophysiology of depression, including the integrity of the blood–brain barrier, gap junctions, glial transmission, glutamate homeostasis and energy metabolism (Liu et al. 2021). Our results indicate the potential ability of EVs to modulate astrocyte subtypes and function. The current mainstream view is that the dysfunction of astrocytes affects the function of neurones in two main ways: the destruction of neurotrophic support and the activation of neurotoxic signalling (Liddelow and Barres 2017). Synaptic plasticity is a fundamental process of brain function that is maintained by astrocytes by regulating the availability of peripheral neurotransmitters and actively driving synaptic formation/elimination (Hasel and Liddelow 2021; Durkee et al. 2021). Substantial evidence indicates that stimulating astrocytes to release endogenous ATP can regulate hippocampal synaptic function, thereby influencing mood and cognition (Cao et al. 2013; Chi et al. 2022; Cho et al. 2022). Conversely, neurotoxic reactive astrocytes can promote neuroplastic deficits through proinflammatory cytokines and reactive oxygen species. In this study, we showed that EVs regulate astrocyte polarization, inhibit the complement C3 pathway, enhance glutathione synthesis, boost hippocampal SOD activity and promote ATP production, collectively conferring neuroprotection. Notably, our proteomic profiling revealed a rich repertoire of proteins involved in antioxidant defence, metabolic regulation and stress response. These components may collectively contribute to the observed attenuation of neurotoxic astrocyte activation and complement C3 downregulation, potentially via direct enzymatic activity or through modulation of intracellular pathways. Although the precise bioactive molecules remain to be fully elucidated, these identified proteins provide compelling candidates for future mechanistic investigations aimed at deciphering the astrocyte‐specific effects of EVs. It is important to note that the present study did not characterize the miRNA content of EVs, which represents a limitation, as cross‐kingdom miRNA transfer is a recognized mechanism by which plant EVs can modulate mammalian cell function.

Traditional intranasal formulations face challenges such as rapid mucociliary clearance and enzymatic degradation, limiting their therapeutic efficacy (Xu et al. 2024). By encapsulating EVs within a thermos‐responsive inulin gel, EVs@IN addresses these limitations through improved mucosal adhesion and controlled release. Unlike synthetic nanoparticles or viral vectors, plant‐derived EVs offer inherent biocompatibility and scalability, aligning with the demand for sustainable biomedical solutions. This approach also circumvents the need for high‐dose systemic drug administration, thereby reducing off‐target effects, a significant drawback of conventional antidepressants (Chen et al. 2023). Furthermore, we have confirmed the potential of this system to load and safely deliver drugs that do not readily cross the blood–brain barrier to brain regions such as the olfactory bulb and hippocampus. This capability significantly expands the therapeutic applications of this delivery system.

While our findings are promising, several limitations should be noted. First, the LPS‐ and CUMS‐induced depression model does not fully capture the complexity of human depression, which involves genetic, environmental and epigenetic factors. Further evaluation in multifactorial and longitudinally stable models is essential to better reflect disease heterogeneity and guide biomarker development for clinical translation. Second, the long‐term safety of repeated intranasal EV administration remains unknown, particularly regarding immune responses or nasal tissue toxicity (Shen et al. 2025). Third, the mechanisms by which EVs modulate astrocyte polarization were not fully elucidated in this study. The precise coupling between astrocyte phenotypic switching and metabolic reprogramming, and how this interplay ultimately drives neuroinflammatory or neuroprotective outcomes, warrants further exploration. Future research can further explore these mechanisms. Additionally, optimizing EV production for scalability and consistency, as well as exploring hybrid hydrogels for enhanced brain targeting, could improve clinical translatability.

In summary, EVs@IN represents an innovative strategy for nasal‐to‐brain delivery, leveraging the unique properties of plant‐derived EVs and inulin gel to achieve sustained release and targeted action. By mitigating neuroinflammation, oxidative stress and synaptic loss, this platform holds significant promise for treating depression and other neuropsychiatric disorders. Further preclinical optimization and clinical validation are essential to advance this approach towards therapeutic application, and this technology may have promising clinical translation potential.

## Author Contributions

**Kangyu Jin**: investigation, data curation, writing–original draft, writing–review and editing. **Ruoxi Wang**: investigation, data curation, writing–original draft. **Bing Chen**: investigation, data curation, writing–original draft. **Danni Zhong**: investigation. **Shangping Cheng**: investigation. **Aiying Tong**: investigation. **Yangjian Qi**: investigation, funding acquisition. **Jing Lu**: funding acquisition, supervision, writing–review and editing, writing–original draft. **Min Zhou**: funding acquisition, supervision, writing–review and editing.

## Funding

This work was supported by the Key R&D Program of China (2022YFA1104900), the Leading Innovative and Entrepreneur Team Introduction Program of Zhejiang (2022R01002), the Natural Science Foundation of Shandong Province (ZR2023ZD30), the Binjiang Institute of Zhejiang University (ZY202205SMKY007) and the National Natural Science Foundation of China (82271561 and 82301708).

## Ethics Statement

All animal procedures were approved by the Animal Experimental Ethical Committee of the First Affiliated Hospital, Zhejiang University School of Medicine (Approval No. 20240680).

## Consent

The manuscript has been submitted with the consent of all authors for publication.

## Conflicts of Interest

The authors declare that they have no conflict of interest.

## Supporting information




**Supplementary Material**: jev270198‐sup‐0001‐SuppMat.docx

**Supplementary Material**: jev270198‐sup‐0001‐SuppMat.xls

## Data Availability

All data relevant to the study are included in the article or uploaded as supplementary information. The raw sequence data have been deposited in the Genome Sequence Archive (GSA: CRA031566) and are publicly accessible at https://ngdc.cncb.ac.cn/gsa. The data that support the findings of this study are available from the corresponding author upon reasonable request.
