# Supplementary material for: Nose‐to‐Brain Delivery of Chlorella vulgaris Extracellular Vesicles for Antidepressant Effects
Source: J Extracell Vesicles. 2025 Nov 18;14(11):e70198. doi: 10.1002/jev2.70198 (PMC12626165; doi:10.1002/jev2.70198)
Supplement: Supplementary file 1 — Supplementary Material: jev270198‐sup‐0001‐SuppMat.docx [file JEV2-14-e70198-s001.docx]

**Nose-to-Brain Delivery of Chlorella Vulgaris Extracellular Vesicles for Antidepressant Effects**

Kangyu Jin *et al.*

Corresponding author: Jing Lu, lujing2016@zju.edu.cn; Min Zhou, zhoum@zju.edu.cn

**Supplemental information**

Figures S1 to S9

Tables S1 and S2


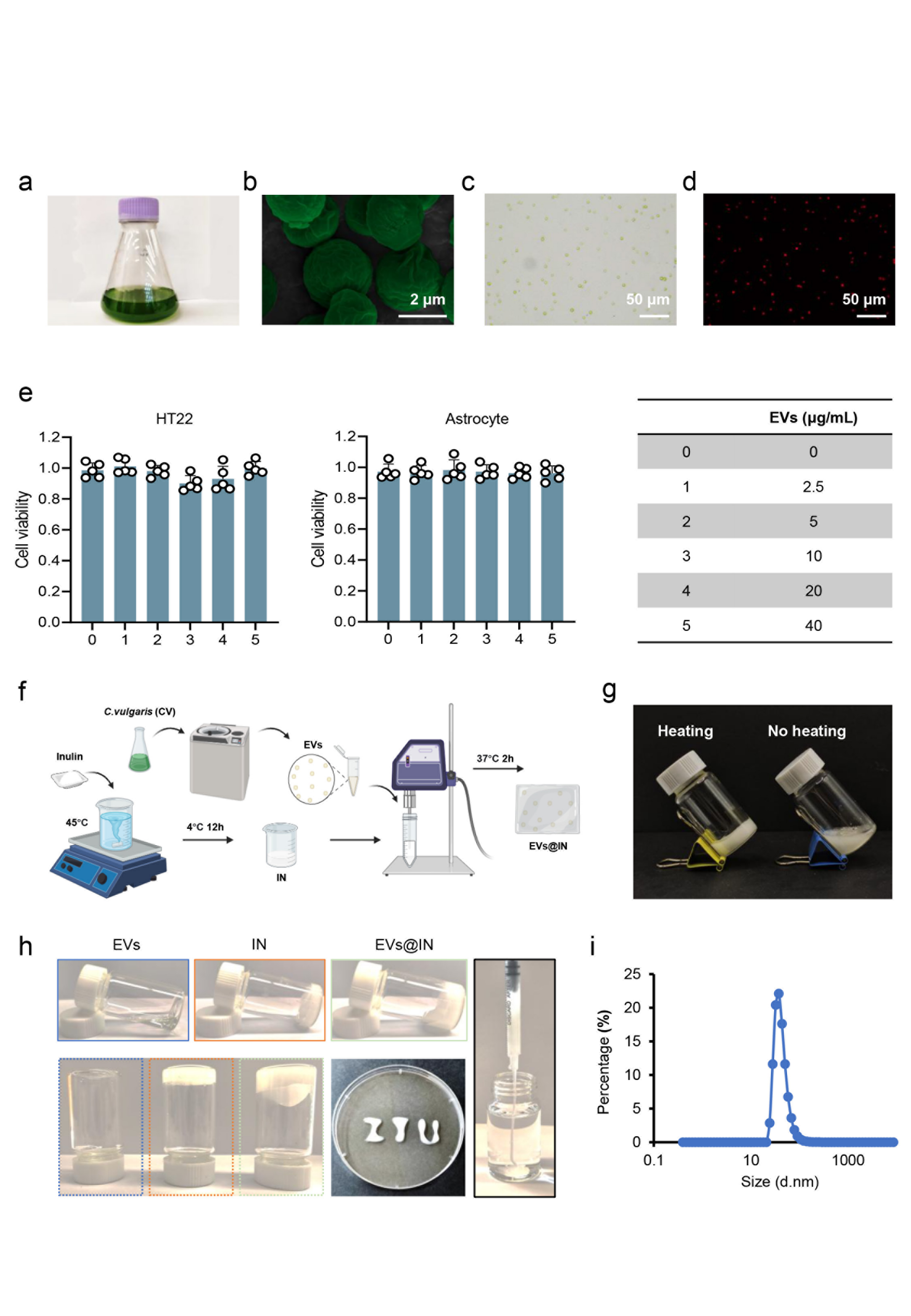


Figure S1: **Fabrication and Characterization of EVs@IN.** (a) a photograph of CV culture under laboratory conditions. (b) SEM image of CV (2–5 µm diameter). Scale bar, 2 μm. (c) Optical microscopy of CV morphology. Scale bar, 50 μm. (d) Fluorescence imaging of CV morphology. Scale bar, 50 μm. (e) Cell viabilities of primary astrocyte cells and HT22 cells after co-culture with different concentrations of EVs for 24 h (n=6). (f) Schematic of EVs@IN synthesis. (g) Effect of heating on IN formation. (h) Fluidity comparison of EVs, IN and EVs@IN. (i) Post-release EVs size analysis by DLS. Data are represented as means ± SEM.


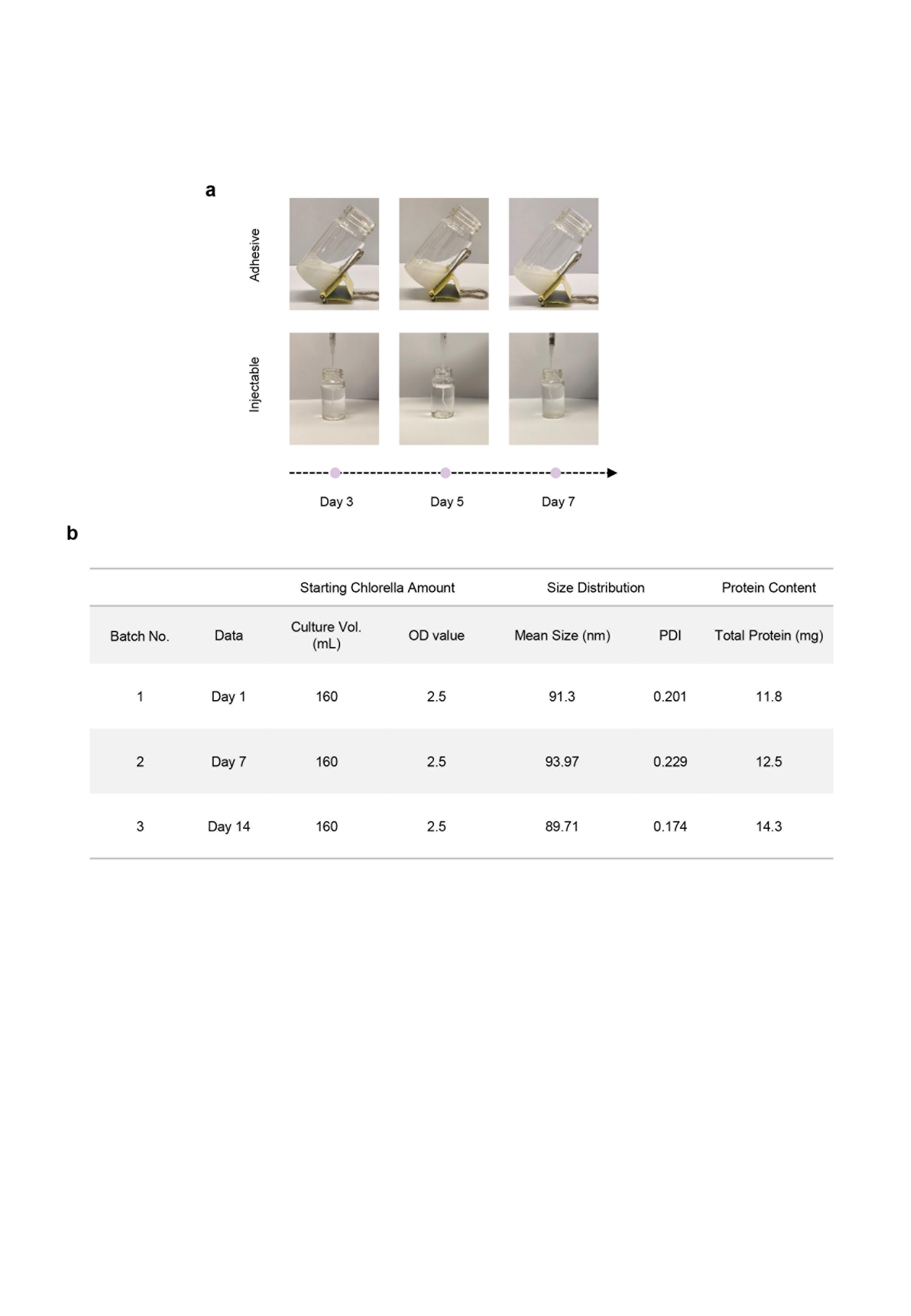
Figure S2: **Storage stability of EVs@IN and batch reproducibility.** (a) Assessment of the adhesiveness (cohesiveness) and injectability (flowability) of the EVs@IN following storage at 4°C for 3, 5, and 7 days. (b) Characterization of three independent batches of EVs, showing the average particle size, polydispersity index (PDI), and total protein content (n=3), confirming the reproducibility of the isolation protocol.


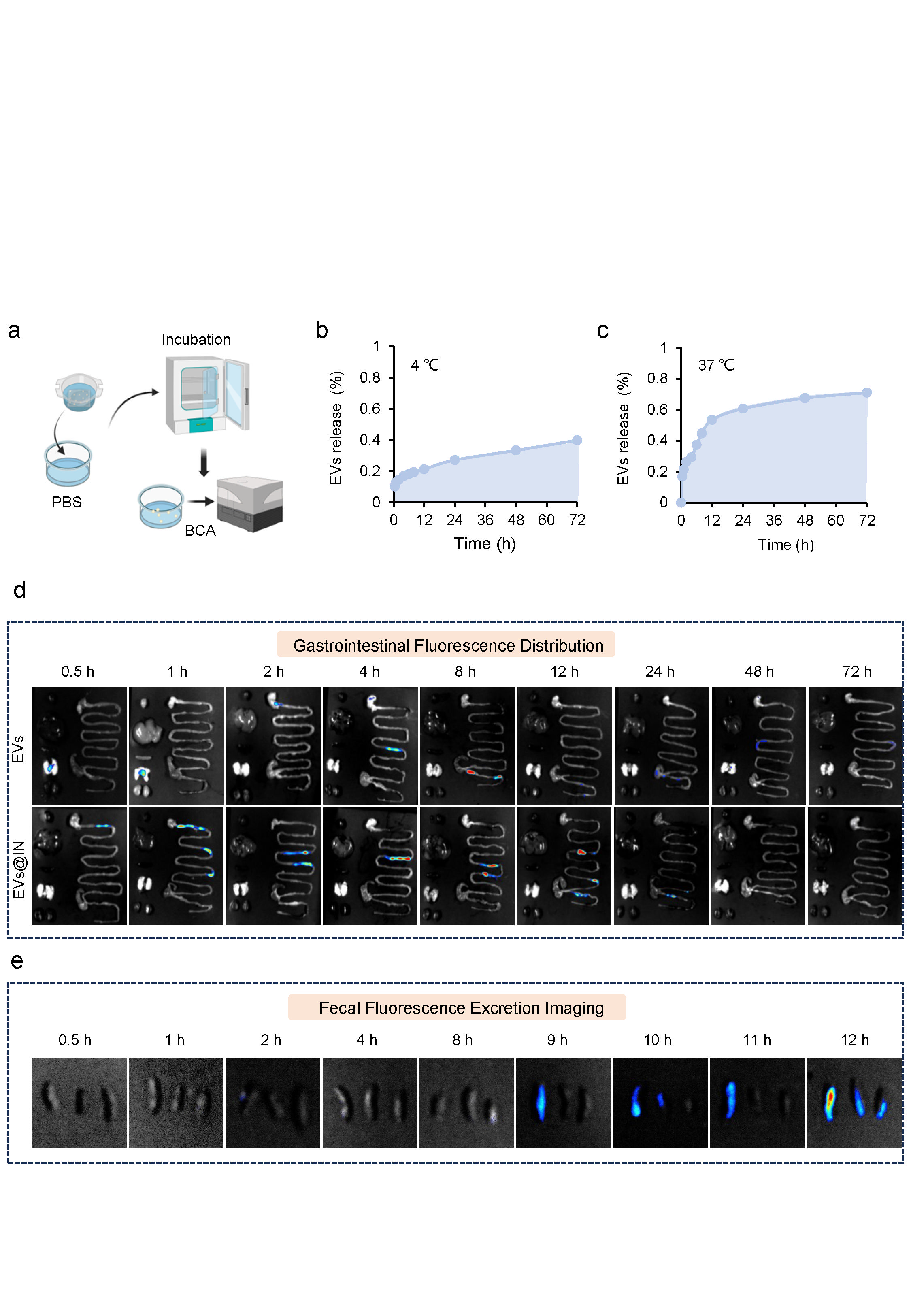


Figure S3: **In vitro release kinetics, biodistribution, and fecal excretion profiles of EVs@IN following intranasal administration.** (a) Schematic of EVs release assay. (b-c) Cumulative EVs release profiles at 4°C and 37°C. (d) Ex vivo fluorescence imaging of major organs at different time points following intranasal administration of EVs and EVs@IN. (e) Time-course ex vivo fluorescence imaging of fecal samples collected from mice treated with EVs@IN. Data are represented as means ± SEM.


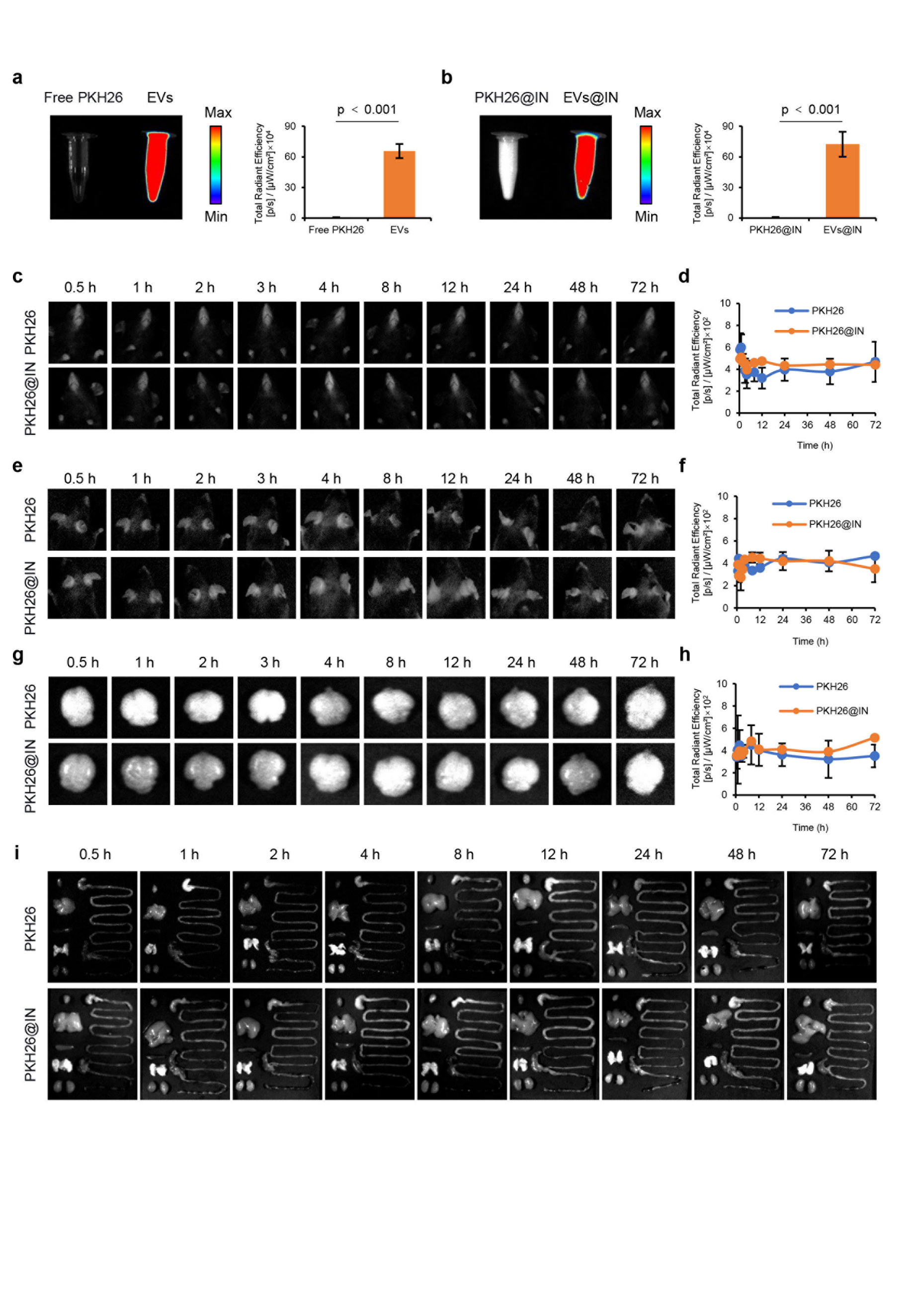


Figure S4: **Control experiments with free dye and dye-loaded hydrogel confirm the specificity of EVs@IN biodistribution**. (a-b) Representative in vitro fluorescence images of free PKH26 dye (PKH26) versus EVs, and PKH26@IN versus EVs@IN. (c) In vivo fluorescence imaging of the nasal region following intranasal administration of PKH26 and PKH26@IN. (d) Quantification of nasal fluorescence signals from (c). (e) In vivo brain fluorescence imaging after administration of PKH26 and PKH26@IN.(f) Quantification of brain fluorescence signals from (e). (g) Ex vivo fluorescence imaging of brains from mice administered of PKH26 and PKH26@IN.(h) Quantification of ex vivo brain fluorescence signals from (g). (i) Ex vivo fluorescence imaging of major organs harvested from mice after administration of PKH26 and PKH26@IN.Data are represented as means ± SEM.


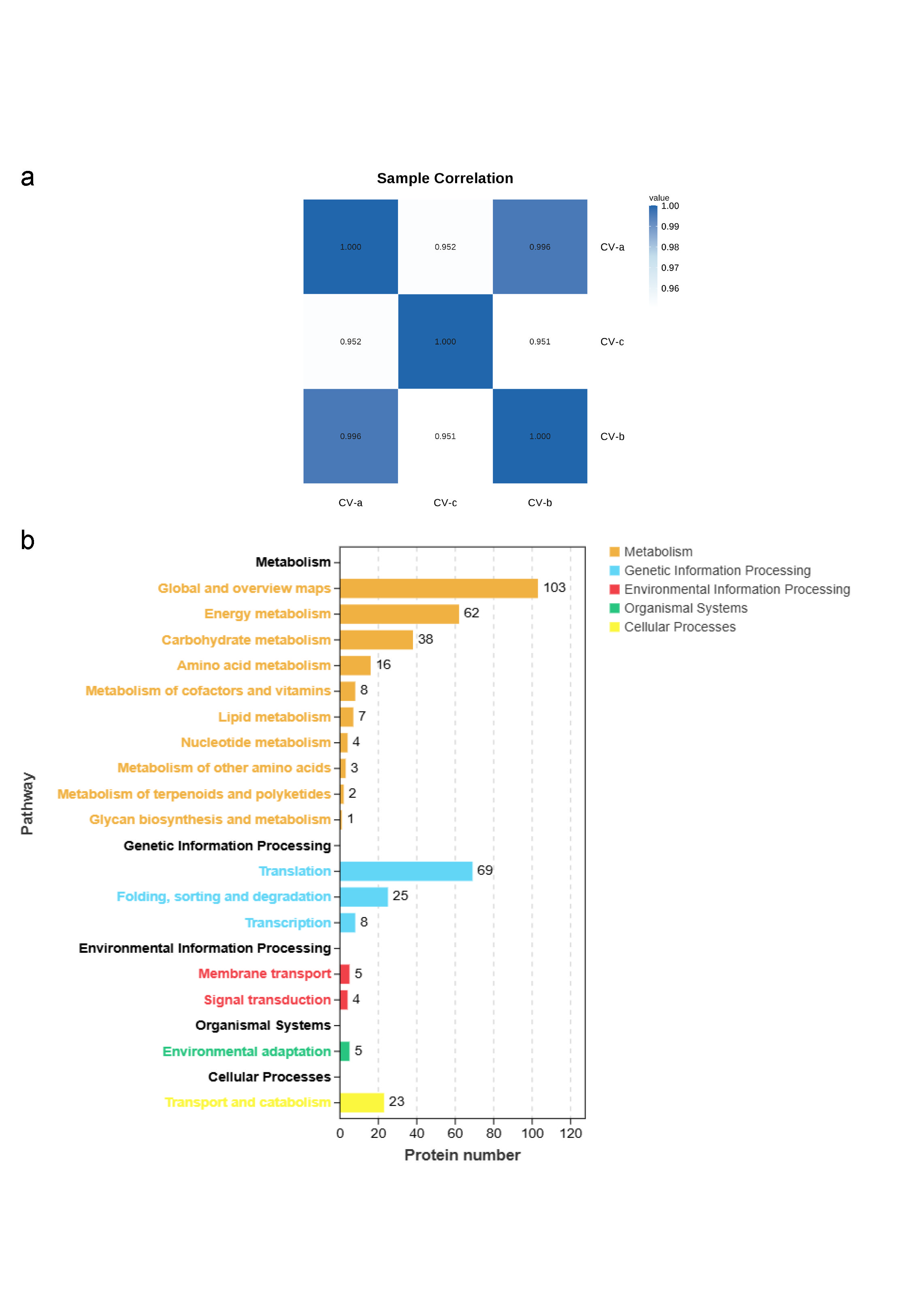


Figure S5: **Characterization of protein components in EVs via proteomic analysis.** (a) Sample similarity analysis. (b) KEGG pathway enrichment analysis.


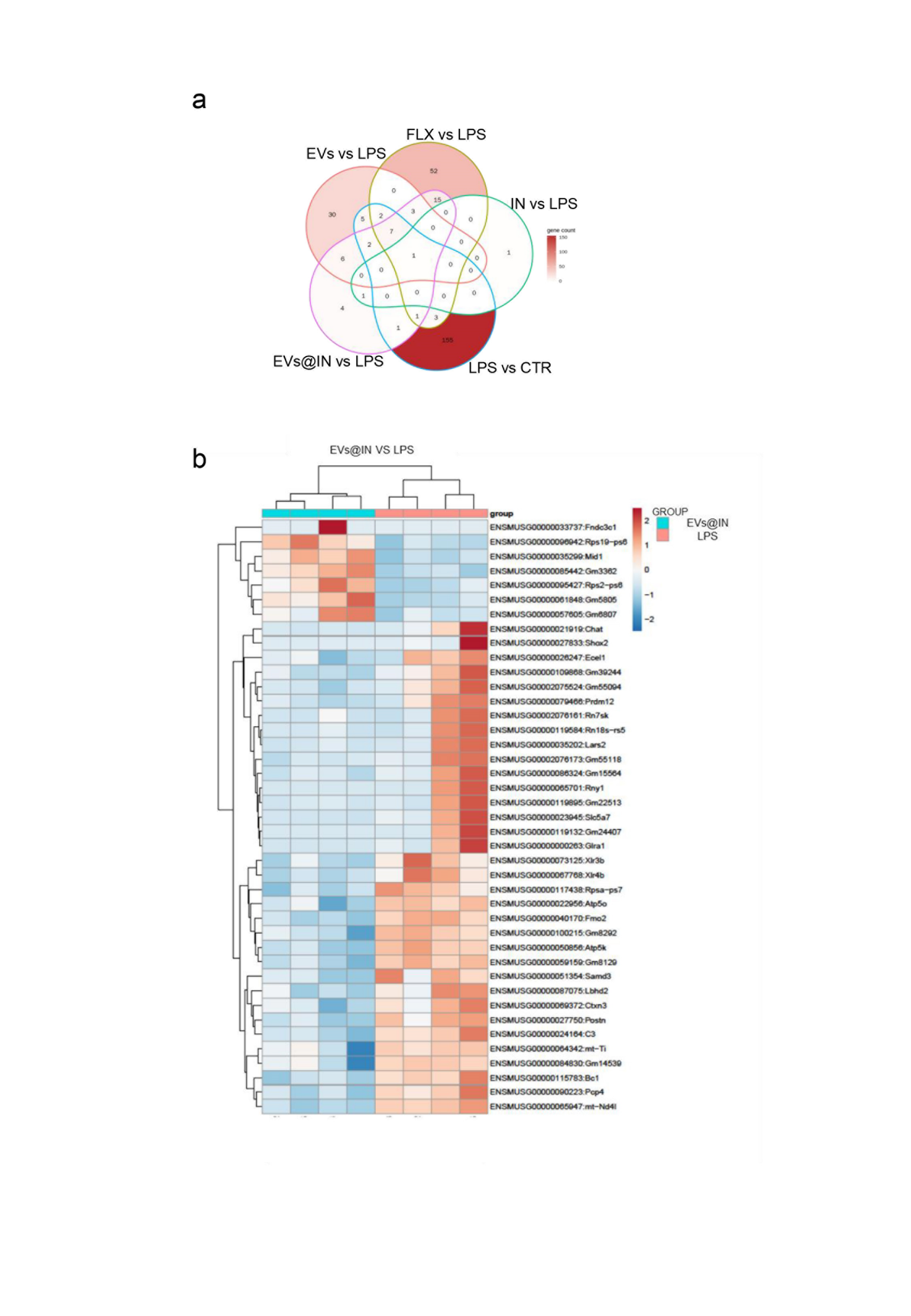


Figure S6: **Heat maps of differential gene expression in different groups.**


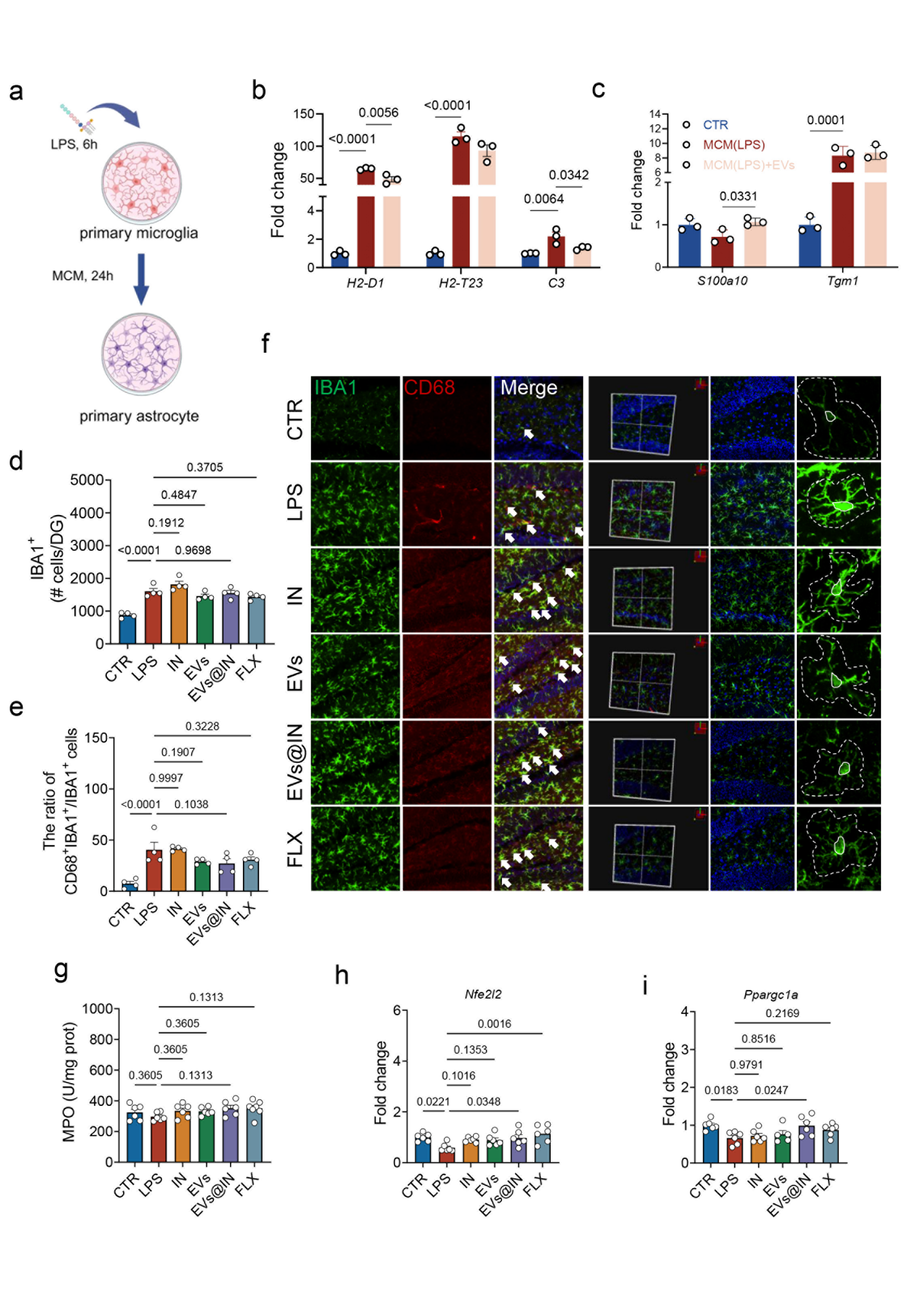


Figure S7: **EVs@IN acts on regulating astrocyte subtype but not microglia**. (a-c) The representative images of CD68^+^IBA1^+^ cells and microglia morphology (a) and the quantification of the ratio of CD68^+^IBA1^+^ cells (b) and the number of IBA1^+^ cells (c) in hippocampus (n=4). (d) myeloperoxidase (MPO) activity in different groups (n=6). (e-f) The mRNA expression of peroxisome proliferator activated receptor γ coactivator-1α (PGC-1α) - nuclear factor erythroid 2-related factor 2 (Nrf2) pathway, respectively (n=6). (g) Schematic of experimental design of the process of treatment with astrocytes. (h-i) The mRNA expression of neurotoxic and neuroprotective astrocytes markers in hippocampal astrocytes in different groups (n=3). Data are represented as means ± SEM. The significance of difference in (b-e) and (g-i) was determined by one-way ANOVA with Dunett's post-hoc test.


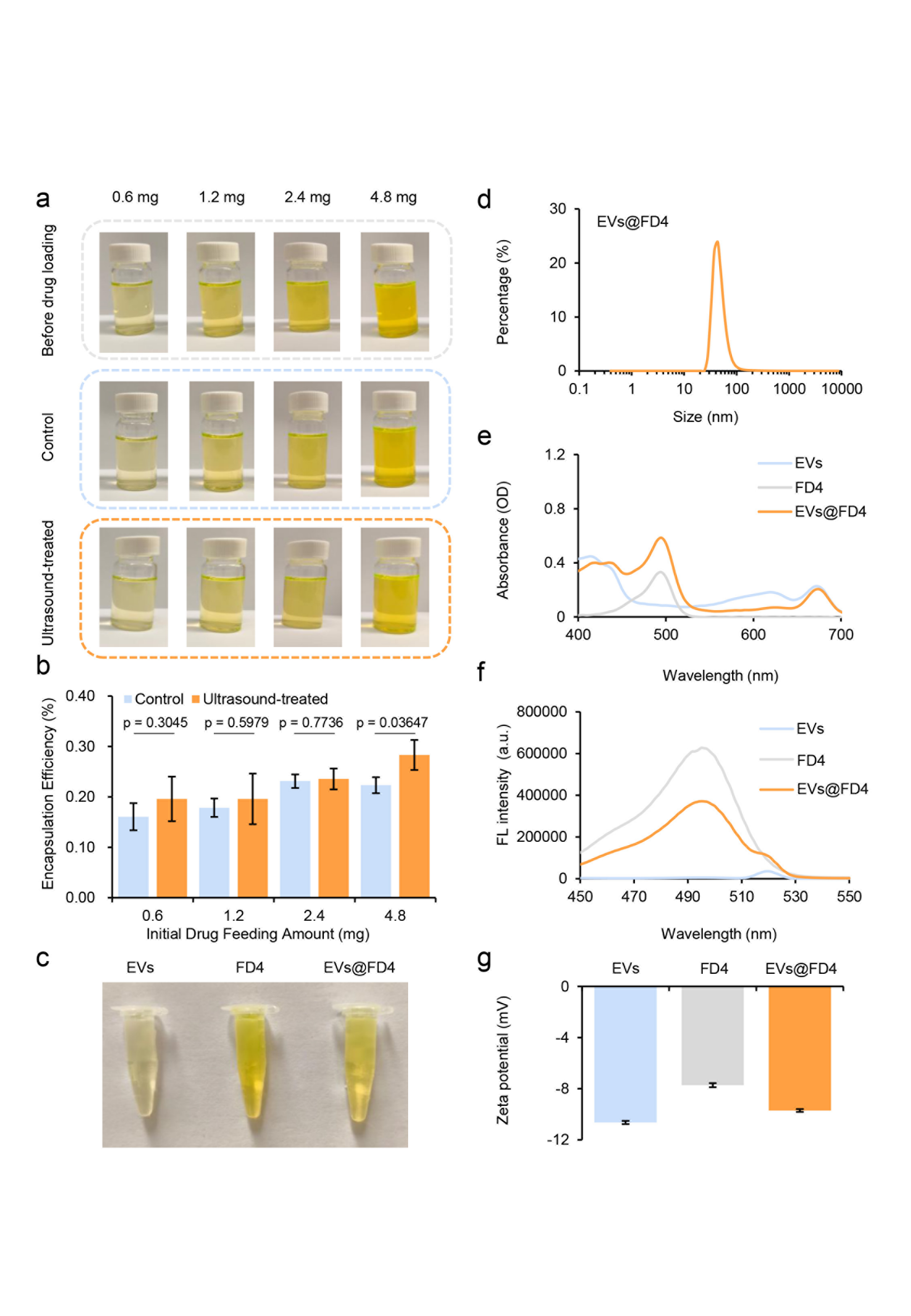


Figure S8: **FD4 encapsulation and characterization of EVs@FD4.** (a) Visual comparison of EVs@FD4 with increasing FD4 doses. (b) Encapsulation efficiency: ultrasound-treated vs. passive loading. (c) Color contrast between EVs, FD4, and EVs@FD4. (d) DLS analysis of EVs@FD4. (e) UV-vis spectra of EVs, FD4 and EVs@FD4. (f) Fluorescence spectra of EVs, FD4 and EVs@FD4. (g) Zeta potential of EVs, FD4 and EVs@FD4. Data are represented as means ± SEM.


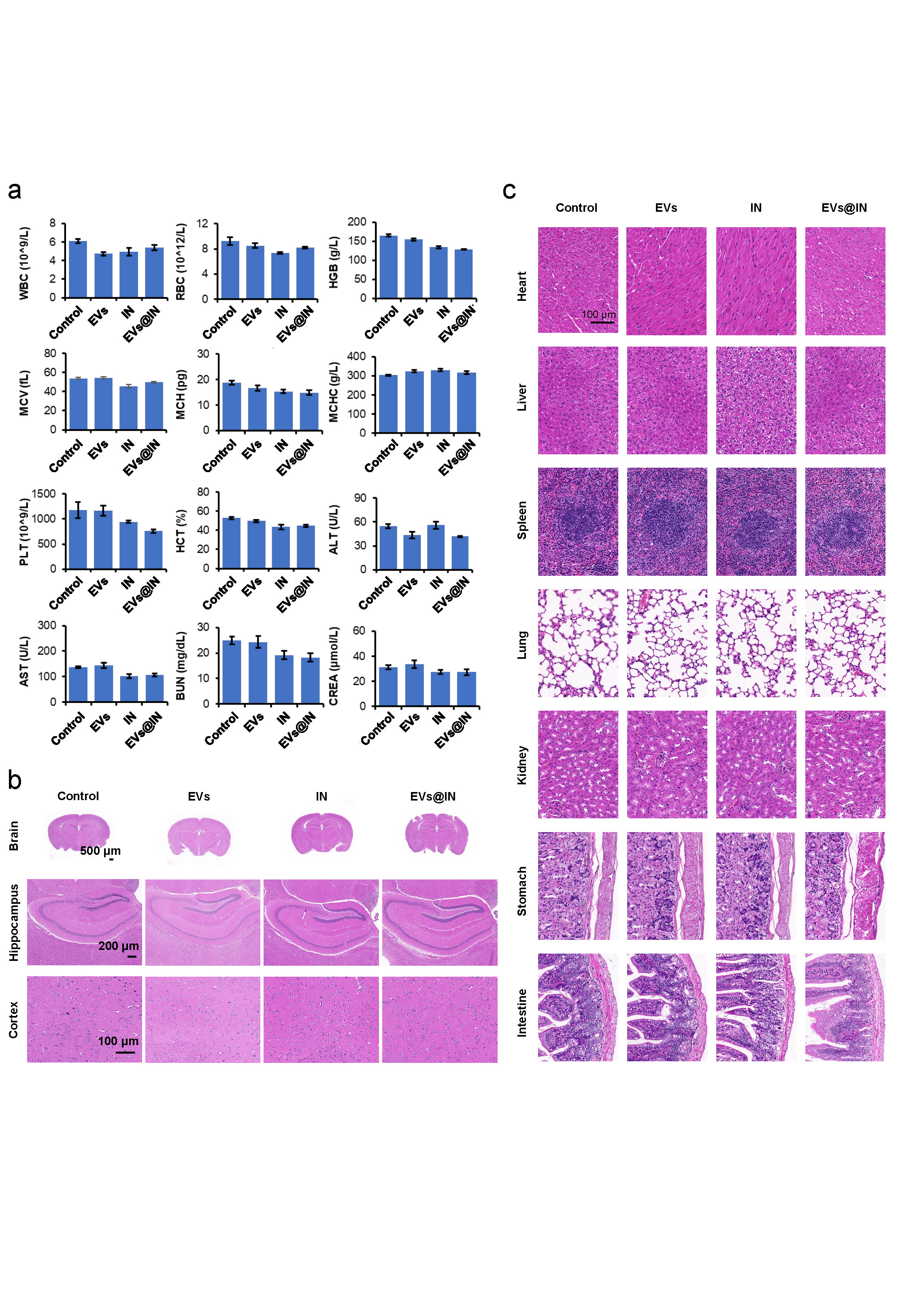


Figure S9: **Biosafety assessment of EVs, IN and EVs@IN in vivo.** (a) Blood routine and biochemical indicators between the treatment groups and the control group. (b) Histopathological examination of brain tissue in different groups. (c) The pathological changes in tissue sections of the heart, liver, spleen, lung, kidney, stomach, and intestine in different groups. Data are represented as means ± SEM.

**Table S1.** Key Resources Table.

| **REAGENT or RESOURCE** | **SOURCE** | **IDENTIFIER** |
| --- | --- | --- |
| **Antibodies** | | |
| Anti-Doublecortin | Abcam | CAT# ab18723  RRID: AB_732011 |
| Anti-NeuN | Cell Signaling Technology | CAT# 24307S  RRID: AB_2651140 |
| Anti-GFAP | Abcam | CAT# ab7260  RRID: AB_305808 |
| Anti-GFAP | Sigma-Aldrich | CAT# SAB2500462  RRID: AB_10603437 |
| Anti-IBA1 | Abcam | CAT# ab178846  RRID: AB_2636859 |
| Anti-PSD95 | Proteintech | CAT# 20665-1-AP  RRID: AB_2687961 |
| Anti-Synaptophysin | Proteintech | CAT# 67864-1-Ig  RRID: AB_2918622 |
| Anti-Component C3 | Affinity | CAT# DF13224  RRID: AB_2846243 |
| Anti-S100a10 | Abcam | CAT# ab76472  RRID: AB_1524359 |
| Anti-Component C3d | R and D Systems | CAT# AF2655  RRID: AB_2066622 |
| Anti-CD68 | Abcam | CAT# ab955  RRID: AB_307338 |
| Fluorescein (FITC)–conjugated Donkey Anti-Goat IgG (H+L) | Proteintech | CAT# SA00003-3  RRID: AB_2857365 |
| CoraLite647-conjugated F(ab')2 Fragment Donkey Anti-Mouse IgG (H+L) | Proteintech | CAT# SA00014-8  RRID: AB_2935621 |
| CoraLite488-conjugated Donkey Anti-Rabbit IgG (H+L) | Proteintech | CAT# SA00013-6  RRID: AB_2890972 |
| CoraLite647-conjugated F(ab')2 Fragment Donkey Anti-Rabbit IgG (H+L) | Proteintech | CAT# SA00014-7  RRID: AB_2935619 |
| Cy5 conjugated Goat Anti-rabbit IgG (H+L) | Servicebio | CAT# GB27303  RRID: AB_2905513 |
| Cy3 Conjugated Donkey Anti-Rabbit IgG (H+L) | Servicebio | CAT# GB21403  RRID: AB_2818951 |
| Cy3-Conjugated Donkey Anti-Goat IgG (H+L) | Servicebio | CAT# GB21404  RRID: AB_2868507 |
| Alexa fluor 488-conjugated Donkey anti-Goat IgG (H+L) | Servicebio | CAT# GB25404  RRID: AB_10815595 |
| **Chemicals and commercial kits** | | |
| FastPure Cell/Tissue Total RNA Isolation Kit V2 | Vazyme | CAT# RC112-01 |
| PerfectStart Green qPCR SuperMix | TransGen | CAT# AQ601-01 |
| HiScript IV All-in-One Ultra RT SuperMix for qPCR | Vazyme | CAT# R433-01 |
| YALEPIC^®^ Stool and Soil Genomic DNA Isolation Kit (RNase-A) | YALI BIOTECH | CAT# YC22008-B |
| Inulin | Sigma-Aldrich | CAT# I2255  CAS: 9005-80-5 |
| TritonX-100 | Sigma-Aldrich | CAT# X100  CAS: 9036-19-5 |
| Phenylmethylsulfonyl fluoride (PMSF) | Sigma-Aldrich | CAT# 52332  CAS: 329-98-6 |
| Bovine Serum Albumin V | Solarbio | CAT# A8020  CAS: 9048-46-8 |
| Immunol Staining Primary Antibody Dilution Buffer | Beyotime | CAT# P0103 |
| Secondary Antibody Dilution Buffer | Beyotime | CAT# P0023D |
| PBS | Solarbio | CAT# P1032  CAS: 12111-21-6 |
| Seahorse XF Cell Mito Stress Test Kit | Agilent | CAT# 103015-100 |
| Seahorse XF DMEM Medium | Agilent | CAT# 103575-100 |
| Seahorse XF Calibrant Solution | Agilent | CAT# 100840-000 |
| Seahorse Cell Culture Plates | Agilent | CAT# 100777-004 |
| Pyruvate | Agilent | CAT# 103578-100 |
| Glutamine | Agilent | CAT# 103579-100 |
| Glucose | Agilent | CAT# 103577-100 |
| poly-D-lysine | Thermo Fisher | CAT# A3890401 |
| Dulbecco's Modified Eagle Medium (DMEM) | BDBio | CAT# L100-500 |
| Dulbecco's Modified Eagle Medium/Nutrient Mixture F-12 (DMEM/F12) | BDBio | CAT# L104-500 |
| fetal bovine serum (FBS) | BDBio | CAT# F801-500 |
| penicillin/streptomycin | Gibco | CAT# 15140122 |
| Superoxide Dismutase (SOD) Activity Assay Kit | Solarbio | CAT# BC0175 |
| Reduced Glutathione (GSH) Content Assay Kit | Solarbio | CAT# BC1175 |
| Myeloperoxidase (MPO) Activity Assay kit | Solarbio | CAT# BC5715 |
| Lactic Acid assay kit | Nanjing Jiancheng Bioengineering Institute | CAT# A019-2-1 |
| Mounting Medium, antifading | Solarbio | CAT# S2110 |
| Lipopolysaccharides | Sigma-Aldrich | CAT# L2880  CAS: 93572-42-0 |
| BCA Protein Assay Kit | Beyotime | CAT# P0009 |
| **Virus strains** |  |  |
| PRV-CAG-3Gc | Brain case | CAT# BC-SL001 |
| NCSP-YFP-2E5 | Brain case | CAT# BC-PRV-801-Plus |
| **Software** |  |  |
| Limelight | Actimetrics | N/A |
| Biorender | Biorender | https://biorender.com/ |
| ImageJ v2.1.0/1.53c | NIH | https://imagej.nih.gov/ij/ |
| Prism v9.5.1 | Graphpad | https://graphpad.com |

**Table S2.** The list of primers used in qRT-PCR.

| **Gene** | **Primer sequences (Forward)** | **Primer sequences (Reverse)** |
| --- | --- | --- |
| *Actb* | AGTCAGACAGCAGAAACTAGAC | ACGATGGATGGGAACACAGC |
| *C1qa* | CTGGCATCCGGACTGGTATC | CTTTCACGCCCTTCAGTCCT |
| *C3* | ATAAAGAGCCAGCGGCTACA | CAGCCGTAGGACATTGGGAG |
| *Gbp2* | CCTGGAACATTCCCTGACCC | AACATAGGTCTGCACCAGGC |
| *H2-D1* | TGGTGCTGCAGAGCATTACA | CACCACAGATGCCCACTTCT |
| *H2-T23* | AGTAAACCTGAGGACCCTGC | TCTGTGAGGCTATGTCATTCGC |
| *Hmga1* | GTCGGGAGTCAGAAAGAGCC | TCCAGTTTCTTGGGTCTGCC |
| *Il1a* | ACGTCAAGCAACGGGAAGAT | AAGGTGCTGATCTGGGTTGG |
| *Nfe2l2* | GCCCTCAGCATGATGGACTT | AACTTGTACCGCCTCGTCTG |
| *Ppargc1a* | GTGTTCTGGTACCCAAGGCA | ATGGTCACCAAACAGCCGAA |
| *Ptx3* | GACCTCGGATGACTACGAGC | CTGCGAGTTCTCCAGCATGA |
| *S100a10* | TGCCATCCCAAATGGAGCAC | CCATGAGCACTCTCAGGTCC |
| *Slc10a6* | GGAGGGCCATGCGAATCTAA | GCAACCAGAGCTTCTGACTCT |
| *Sphk1* | CCTGGGCAACACCGATAAGA | CTGGTTCCATAGCCAGGTCC |
| *Tgm1* | GGCAGGTACTAGCCAAGCAA | GTGCACTTGGGAAAGCTGTG |
| *Tnf* | AGTGTTCCCACACCTCTCTCT | CCCAGCAAGCATCTATGCAC |
| *Tubb6* | GGAGCCCTACAATGCCACAT | GGTTGAGGTCCCCGTAAGTG |

Actb, actin, beta; C1qa, complement component 1, q subcomponent, alpha polypeptide, also C1q; C3, complement component 3; Gbp2, guanylate binding protein 2; H2-D1, histocompatibility 2, D region locus 1; H2-T23 histocompatibility 2, T region locus 23; Hmga1, high mobility group AT-hook 1; Il1a, interleukin 1 alpha; Nfe2l2, nuclear factor, erythroid derived 2, like 2, also Nrf2; Ppargc1a, peroxisome proliferative activated receptor, gamma, coactivator 1 alpha, also Pgc-1alpha; Ptx3, pentraxin related gene; S100a10, S100 calcium binding protein A10; Slc10a6, solute carrier family 10 (sodium/bile acid cotransporter family), member 6; Sphk1, sphingosine kinase 1; Tgm1, transglutaminase 1, K polypeptide; Tnf, tumor necrosis factor, also TNF-alpha; Tubb6, tubulin, beta 6 class V.
